# Transfer of patient’s peripheral blood mononuclear cells (PBMCs) disrupts blood–brain barrier and induces anti-NMDAR encephalitis: a study of novel humanized PBMC mouse model

**DOI:** 10.1186/s12974-023-02844-4

**Published:** 2023-07-13

**Authors:** Yaqing Shu, Fuhua Peng, Bingchu Zhao, Chunxin Liu, Qihui Li, Huilu Li, Yuge Wang, Yanjun Jiang, Tingting Lu, Qin Wang, Jian Sun, Huiyu Feng, Zhengqi Lu, Xiaodong Liu, Jie Wang, Wei Qiu

**Affiliations:** 1 Department of Neurology, The Third Affiliated Hospital of Sun Yat-Sen University, Guangzhou, 510630 China; 2 Key Laboratory of Magnetic Resonance in Biological Systems, State Key Laboratory of Magnetic Resonance and Atomic and Molecular Physics, National Center for Magnetic Resonance in Wuhan, Wuhan Institute of Physics and Mathematics, Innovation Academy for Precision Measurement Science and Technology, Chinese Academy of Sciences-Wuhan National Laboratory for Optoelectronics, Wuhan, 430071 China; 3 Department of Emergency, The Third Affiliated Hospital of Sun Yat-Sen University, Guangzhou, China; 4 Department of Anaesthesia and Intensive Care, Faculty of Medicine, The Chinese University of Hong Kong, Hong Kong SAR, China; 5 Li Ka Shing Institute of Health Science, The Chinese University of Hong Kong, Hong Kong SAR, China; 6 Peter Hung Pain Research Institute, The Chinese University of Hong Kong, Hong Kong SAR, China; 7 Department of Pharmacology, Zhongshan School of Medicine, Sun Yat-Sen University, Guangzhou, China; 8 Guangdong Laboratory for Lingnan Modern Agriculture, National Risk Assessment Laboratory for Antimicrobial Resistance of Animal Original BacteriaCollege of Veterinary Medicine, South China Agricultural University, Guangzhou, China; 9 Department of Neurology, The First Affiliated Hospital of Sun Yat-Sen University, Guangzhou, China; 10 Institute of Neuroscience and Brain Diseases; Xiangyang Central Hospital, Affiliated Hospital of Hubei University of Arts and Science, Xiangyang, Hubei China; 11 University of Chinese Academy of Sciences, Beijing, 100049 China

**Keywords:** Humanized mouse model, Anti-*N*-methyl-d-aspartate receptor (NMDAR) encephalitis, Peripheral blood mononuclear cells (PBMC), Blood–brain barrier (BBB), Il-1β, Anakinra

## Abstract

**Background:**

Anti-N-methyl-D-aspartate receptor (NMDAR) encephalitis is a severe autoimmune neuropsychiatric disease. Brain access of anti-NMDAR autoantibody through the blood–brain barrier (BBB) is essential for pathogenesis. Most previous animal models limit the investigation of etiologies of BBB damage in patients.

**Methods:**

In this study, we established a novel humanized mouse model of anti-NMDAR encephalitis by intraperitoneal injection of patients’ peripheral blood mononuclear cells (PBMCs) into BALB/c *Rag2*^−/−^*Il2rg*^−/−^*Sirpα*^NOD^*Flk2*^−/−^ mice.

**Results:**

We found that engraftment of patients’ PBMCs not only produced potent anti-GluN1 autoantibodies, but also disrupted BBB integrity to allow brain access of autoantibodies, resulting in a hyperactive locomotor phenotype, anxiety- and depressive-like behaviors, cognitive deficits, as well as functional changes in corresponding brain regions. Transcriptome analysis suggested an exaggerated immune response and impaired neurotransmission in the mouse model and highlighted Il-1β as a hub gene implicated in pathological changes. We further demonstrated that Il-1β was produced by endothelial cells and disrupted BBB by repressing tight junction proteins. Treatment with Anakinra, an Il-1 receptor antagonist, ameliorated BBB damage and neuropsychiatric behaviors.

**Conclusions:**

Our study provided a novel and clinically more relevant humanized mouse model of anti-NMDAR encephalitis and revealed an intrinsic pathogenic property of the patient’s lymphocytes.

**Supplementary Information:**

The online version contains supplementary material available at 10.1186/s12974-023-02844-4.

## Introduction

Anti-N-methyl-D-aspartate receptor (NMDAR) encephalitis is a rare severe autoantibody-mediated autoimmune neuropsychiatric disease that commonly presents with behavioral changes, psychosis, memory impairment, and seizures [1, 2]. The disorder is characterized by circulating anti-NMDAR autoantibodies (NMDAR-Abs), which causes a selective and reversible internalization of cell-surface NMDARs in cultured neurons [3]. Although tumorigenesis and viral infection are potentially two major causes [4], the etiology and pathogenesis of anti-NMDAR encephalitis are still largely unknown.

Animal models, particularly mouse models, are powerful research tools that advance the understanding of disease pathogenesis. To date, several types of mouse models for anti-NMDAR encephalitis have been established, including antibody passive transfer mouse models [5, 6] and active immunization mouse models [7, 8]*.* However, these mouse models have been limited by practical considerations, genetic differences, or species differences. For example, although passive anti-NMDAR antibodies’ transfer can cause hypofunction in NMDAR-mediated synaptic transmission [6], these models fail to recapitulate the immunological factors and lack the feature of immune cell infiltration and neuroinflammation in the disease process [9, 10]. Active immunization with NMDAR peptides/proteins elicits strong immune responses that produce anti-NMDAR antibodies and initiate brain infiltration of immune cells in mice [8]. However, these models may neglect the etiology of the autoimmune response and etiology-associated immunopathology in humans. In this regard, the establishment of humanized mice with patients’ pathophysiological systems may partially compensate for the shortcomings of previous models [11].

Transfer of patient-derived peripheral blood cells into mice with severe combined immunodeficiency (SCID) represents a widely accepted humanized mouse model that has long been used to investigate autoimmune diseases, such as Myasthenia gravis (MG) [12], idiopathic nephrotic syndrome [13], systemic lupus [14, 15], systemic sclerosis (SSc) [16, 17], and Rasmussen’s encephalitis [18]. BALB/c *Rag2*^−/−^*Il2rg*^−/−^*Sirpα*^NOD^*Flk2*^−/−^ mice (also known as BRGSF mice) are typical SCID mice that lack functional B, T, and NK cells while being highly permissive to the engraftment of human peripheral blood mononuclear cells (PBMCs), due to SIRPα^NOD^ expression. BRGSF mice have become the most popular and instrumental tools for studying the in vivo immunobiology of human lymphoid cells in response to various environmental challenges [19–21].

In the current study, we reported that engraftment of PBMCs from patients with anti-NMDAR encephalitis into BRGSF mice could produce the core clinical phenotypes and closely reflect the characteristics of the human disease in mice. Furthermore, we identified a pathogenetic role of altered endothelial cells in the blood–brain barrier (BBB) using this humanized mouse model and reported endothelium-derived Interleukin 1β (Il-1β) as a therapeutic target for anti-NMDAR encephalitis.

## Materials and methods

### Patients and healthy subjects

Anti-NMDAR encephalitis was diagnosed according to the guidelines [22, 23]. A total of 13 patients with anti-NMDAR encephalitis and 13 healthy control subjects (HC) of similar age and sex were enrolled in the Department of Neurology, The Third Affiliated Hospital of Sun Yat-sen University, Guangzhou, China. The demographic and clinical characteristics of the subjects are summarized in Additional file 1: Tables S1 and S2. PBMCs were collected from all patients and HC, and the mRS scores (severity of disease) were evaluated when the patients were initially admitted and at the period of discharge. In total, 18 PBMC samples (*n* = 9 for each group, Additional file 1: Table S1) were transferred into BRGSF mice (Additional file 1: Table S3); the detailed numbers of recipient mice for each donor of PBMCs are provided (Additional file 1: Table S3). In total, 10 PBMC samples (*n* = 5 for each group) were used in experiment in vitro (Additional file 1: Table S2). Note that PBMCs from patient #4 and HC 7 were used in both in vivo and in vitro experiments (Additional file 1: Table S2), and there was no significant difference between the clinical characteristics of patients used for animals and in vitro experiments (Additional file 1: Table S4).

This study was performed in accordance with the 1964 Helsinki Declaration and approval was obtained from the Institutional Ethics Committee of The Third Affiliated Hospital of Sun Yat-sen University ([2019]02-637-01). All participants agreed and signed the written informed consent.

### Mice

**Fig. 1:**
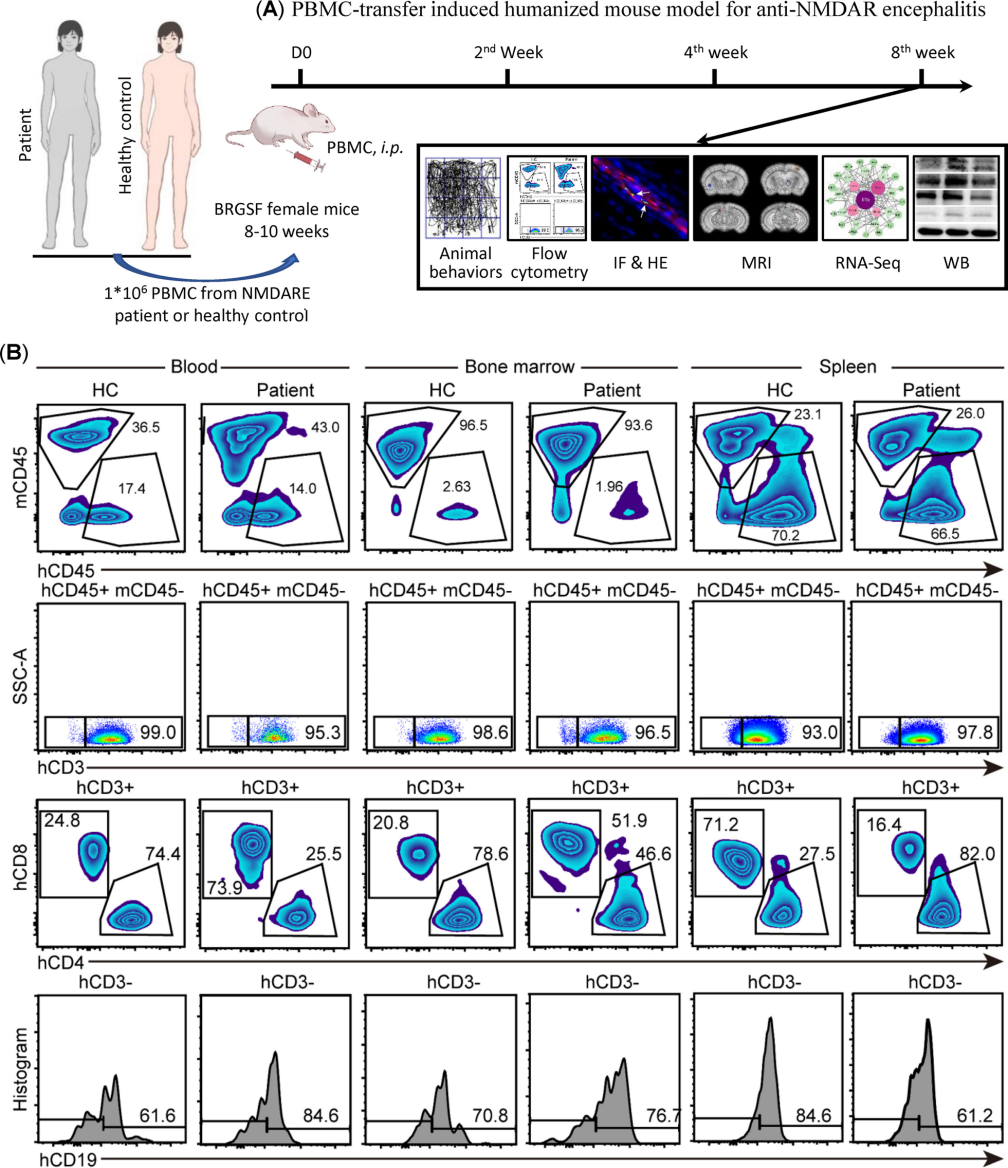
Illustration of the experimental design and verification of PBMC engraftment in BRGSF mice. **A** Schematic representations of the experimental design. **B** Representative images of flow cytometry for mouse (m) CD45^+^, human (h) CD45^+^, hCD3^+^, hCD4^+^, hCD8^+^, and hCD19^+^ cells in blood, bone marrow, and spleen of BRGSF mice. HC and patient refer to mice injected with PBMCs from healthy control and patients (*n* = 3/group), respectively

The schematic diagram of the experimental design is shown in Fig. 1A. Female BRGSF mice, 8–10 weeks, were purchased from Cyagen Biosciences (Suzhou, Jiangsu, China) and housed under specified pathogen-free conditions, with 12-h light/darkness cycles, at the animal facility of South China Agricultural University. Mice were randomly assigned to three groups and injected with cell medium (NC group), PBMCs from HC (HC group), or PBMCs from patients with anti-NDMAR encephalitis (patient group).

### Human PBMC isolation and transfer

Two batches of PBMCs were collected for animal and in vitro studies. The PBMCs adopted in the animal studies (Additional file 1: Table S1) and in the in vitro study (Additional file 1: Table S2) were collected from subjects who met the enrollment criteria and signed the consent form during hospital admission from 2020-07-07 to 2022-05-12. There are no additional criteria for selecting PBMC samples for animal studies. All patient blood was collected during hospital admission. The demographic and clinical characteristics of the subjects were compared between two batches (Additional file 1: Tables S1 and S2, and Additional file 2).

Venous blood was collected in the morning after an overnight fast; then, one part of venous blood was isolated by centrifugation at 3000 rpm for 10 min within 1 h of sample collection. The separated serum was stored at − 80 °C before evaluation for detecting anti-NMDAR abs. Another part of venous blood was used for PBMC isolation. Lumbar punctures were performed under standardized conditions at the L3–L4 or L4–L5 interspace. Cerebrospinal fluid (CSF) samples were collected, immediately aliquoted, and frozen at – 80 °C until analysis. All patients’ serum and CSF were analyzed by indirect immunostaining using transfected human embryonic kidney cells (HEK293) expressing NR1 subunits of the NMDA receptor, as described in our previous work [23].

PBMCs were isolated using a PBMC isolation kit (LTS1077, TBD Science, China) according to the manufacturer’s instructions. Whole blood (5 mL) in ethylenediaminetetraacetic acid (EDTA) anticoagulant tube was mixed with 5 mL of PBS and slowly added to 50 mL centrifuge tube containing 10 mL isolation solution. PBMCs were obtained by density gradient centrifugation at 400×*g* for 30 min at 20 °C. After isolation, PBMCs were resuspended in cryopreservation solution with 10% DMSO and subsequently transferred to 2 mL cryovials and frozen at − 80 °C in an NALGENE TM Cryo 1 °C freezing container (Nalgene, Waltham, MA, USA) overnight. Afterward, frozen PBMCs were stored in liquid nitrogen.

For cell transfer, PBMCs (1 × 10^6^ in 100 μL RPMI 1640 medium) from healthy subjects or patients with anti-NMDAR encephalitis were injected intraperitoneally into each BRGSF mouse [18]. Each mouse received only one injection of PBMCs. Mice in the negative control (NC) group were injected with RPMI 1640 medium. Whole blood, cerebrospinal fluid (CSF), bone marrow, spleens, and brains were collected 8 weeks after transplantation at the end of the experiment. To collect CSF and blood samples from animals, mice were first anaesthetized with 3% isoflurane for induction. Afterward, the mice were maintained under anesthesia with 1.5% isoflurane and CSF was collected using a cisterna magna puncture technique, according to a previously described method [24]. Blood samples were collected from tail veins.

### Behavioral assessments

Behavioral assessments were conducted 8 weeks after PBMC transfer by a researcher who was blind to the treatment conditions.

#### Elevated plus maze (EPM)

The device consists of two closed (35 × 5 × 15 cm^3^) and two open (30 × 6 × 0.6 cm^3^) arms that extend from the same central platform (6 × 6 cm^2^). Each mouse was placed in the center of an open arm and videotaped for 10 min. The time spent in open and closed arms was analyzed using the DigBehv software.

#### Novel object recognition test (NORT)

NORT was performed to evaluate the recognition memory of mice. First, the mice were allowed to explore the open box (50 × 50 × 40 cm^3^) for 10 min. After a period of 24 h, two identical objects were placed in opposite corners of the open box. A mouse was placed in the center of the open box, followed by recording exploration times on the two objects (a1 and a2) over a 10 min period. Two hours later, one of the objects was replaced with a novel object. The mouse was allowed to explore freely for another 10 min, and the exploration time on the familiar object (a) and the novel object (b) was recorded. The mice that did not achieve a minimum of 20 s of exploration for any of the objects were excluded from the analysis. The recognition index was calculated as Time_b_/(Time_a_ + Time_b_) × 100.

#### Forced swimming test (FST)

Mice were individually placed in a transparent cylinder (10 cm in diameter) with water (maintained at 24 ± 1 °C) at a depth of 16 cm. A video camera mounted over the cylinder and the immobilization time of a mouse was recorded using a tracking system to assess depressive-like behaviors. The immobilization time was defined as the time when the animal stopped struggling and started floating in water. The recording time for a single trial was 6 min, and the measurement of the immobilization time began from the second minute.

#### Open-field test (OFT)

The mice were placed in the center of a 50 × 50 × 40 cm^3^ box and allowed to move freely while being recorded for a period of 10 min. The DigBehv software (Shanghai Jiliang Software Technology Co. Ltd, China) was used to track the total distance traveled. This total distance and the time in the central area (CA) were used to assess the overall level of locomotor activity and anxiety, respectively.

### Magnetic resonance imaging (MRI)

#### MRI data acquisition

Magnetic resonance imaging (MRI) was performed on mice using a 9.4 T small-animal scanner (BioSpec94/30 USR; Bruker, Karlsruhe, Germany). The animals were anesthetized with isoflurane and placed on the animal bed. The animal’s body temperature (~ 36.5 °C) was maintained with a circulating warm water pad under the animal bed. The respiratory rate was monitored and kept at around 70 breaths/min by adjusting the isoflurane concentration during acquisition of MRI data. Brain volumes of animals were assessed using a high-resolution morphological T2-weighted TurboRARE sequence, and the parameters were set as follows: repetition time/echo time = 4000/32 ms, 7 averages, rapid acquisition with relaxation enhancement [RARE] factor = 8, 38 coronal slices per 0.4 mm, field of view (FOV) = 15 × 15 mm^2^, matrix = 192 × 192, in-plane resolutio*n* = 0.078 × 0.078 mm^2^. Next, the functional MRI (fMRI) data were collected with gradient-echo echo-planar imaging (GE EPI) pulse sequence for the calculation of the amplitude of low-frequency fluctuations (ALFF). The fMRI data were acquired using the following parameters: TR (repetition time) = 2000 ms, TE (echo time) = 9.05 ms, matrix size = 75 × 75, FOV = 15 × 15 mm^2^, slice number = 40, slice thickness = 0.4 mm, in-plane resolutio*n* = 0.2 × 0.2 mm^2^, no gap. Each resting-state fMRI was acquired for 5 min and included 150 frames.

#### MRI data analysis

All MRI/fMRI data were preprocessed with AFNI (National Institutes of Health, USA), FSL (www.fmrib.ox.ac.uk/fsl/), and ANTs (http://stnava.github.io/ANTs/). The scalp and skull tissue were manually removed from the MRI data. For the fMRI data, the mice brain imaging data were preprocessed with the following steps: time despiking, slice timing, motion correction, nuisance regression (6 affine motion parameters were regressed from the data set), temporal filtering with a bandpass filter (0.01–0.1 Hz), and spatial smoothing performed with a Gaussian kernel (FWHM = 0.4 mm).

For the volume calculation, the standard template was registered to the T2 imaging using the registrationSyN function in the software ANTs; then, the atlas of the standard template was transformed using the transformation matrix generated by the standard template based on the antsApplyTransforms function in ANTs. Finally, the Python-based nibabel function was utilized to count the volume sizes of different brain regions voxel by voxel.

For ALFF analysis, the first 10 images of each mouse were discarded to allow magnetization equilibrium. The 11th EPI image was registered to the T2 image, which was then registered to the standard template to generate 2 transformation matrices for subsequent calculation. Finally, the antsApplyTransforms function was used to register the EPI images of different mice to a standard template and calculate the ALFF values using the 3dRSFC command in AFNI.

### Flow cytometry

Leukocytes from mice blood, spleen, and bone marrow (BM) were quantified using flow cytometry. Briefly, 100 μL peripheral blood was collected in EDTA anticoagulant tubes. Single-cell suspensions of the spleen were obtained through mechanical disaggregation. Single-cell suspensions of the BM were flushed from femurs and tibias. These single-cell suspensions were then filtered through 70 μm cell strainers (15-1070, Biologix, West Hollywood, CA, USA). The erythrocytes were lysed using RBC lysis buffer (R1010, Solarbio, Beijing, China). After 2 washes with staining buffer, cells were preincubated with Human TruStain FcX™ (422302, Biolegend, San Diego, CA, USA) for 10 min at room temperature, followed by staining with fluorochrome-labeled antibodies (1:50) in the dark at 4 °C for 45 min, and detection with an LSRfortessa flow cytometer (BD Biosciences, San Jose, CA, USA). The following fluorochrome-conjugated antibodies were used: PE-Cy™5 anti-human CD45 (555490, BD Biosciences), PE anti-mouse CD45 (561087, BD Biosciences), APC R700 anti-human CD3 (659119 BD Biosciences), APC anti-human CD4 (565994, BD Biosciences), FITC anti-human CD8 (555634, BD Biosciences), BV421 anti-human CD19 (562440, BD Biosciences). The data were analyzed employing FlowJo software.

### Hematoxylin and eosin (H&E) staining

Paraffin-embedded tissues were cut into 3 μm sections. H&E staining was performed according to the standard protocol. Briefly, sections were treated as follows: dewaxing (xylene, 2 × 2 min), rehydration (90% ethanol for 10 min, 80% ethanol for 10 min, and 75% ethanol for 10 min), hematoxylin staining (5 min, G1004, Servicebio, Wuhan, Hubei, China), differentiation (2 s, G1039, Servicebio), bluing (2 s, G1040, Servicebio), dehydration (85% ethanol for 5 min and 95% ethanol for 5 min), eosin staining (5 min, G1001, Servicebio), dehydration (anhydrous ethanol for 3 × 5 min), and hyalinization (xylene, 2 × 5 min). Sections were then sealed with neutral gum (10004160, Sinopharm, Beijing, China) and photographed using an Olympus IX-71 inverted fluorescent microscope (Olympus, Tokyo, Japan).

### Immunohistochemistry

Paraffin-embedded section (3 µm thick) were dewaxed in xylene and heated in 0.01 M sodium citrate buffer (pH = 6.0) in a microwave at 60 °C for 20 min for antigen retrieval. Tissue sections were then blocked in 0.3% H_2_O_2_ for 10 min to remove endogenous peroxidase activity, followed by incubation with primary antibody against CD4 (1:1000; ab288724, abcam, Cambridge, MA, USA), CD20 (1:50; ab78237, abcam), or CD138 (1:100; ab130405, abcam). The target was visualized with a DAB staining kit (GK600710, Gene Tech, Shanghai, China).

### Detection of anti-NMDAR autoantibodies

The presence of human anti-NMDAR antibodies in the serum of mice was determined using immunofluorescence staining of GluN1-transfected cells. An amount of 1 × 10^5^ HEK-293 T cells in 24-well plates on 12 mm cover-slips were transfected with 500 ng M68CT–GluN1 plasmid using 1 μL Lipofectamine™ 3000 reagent (L3000015, Invitrogen, Grand Island, NY, USA). After 48 h, cells were fixed with 4% paraformaldehyde for 30 min at room temperature, blocked with 5% BSA–0.3% Triton ™ X-100 (V900502, Vetec, Green Lane, PA, USA) for 1 h at room temperature, and then incubated with mouse serum (1:1) at 4 °C overnight and with Alexa Flour 488-conjugated goat anti-human IgG (1:500; A-11013, Invitrogen) at room temperature for 1 h. Afterward, the cells were incubated again with commercial rabbit anti-GluN1 antibody (1:200; AGC-001, Alomone, Jerusalem, Israel) at 4 °C overnight and then with Alexa Flour 555-conjugated goat anti-rabbit IgG (1:500; 4417, CST, Novi, MI, USA) at room temperature for 1 h. Cell nuclei were counterstained with DAPI (1:2000; Sigma, Cambridge, MA, USA) for 10 min at room temperature, followed by sealing and photographing using an inverted fluorescence microscope Olympus IX-71 (Olympus).

### Dot blot

A nitrocellulose membrane premoistened with tris-buffered saline (TBS) was placed on a Bio-Dot apparatus (706545, Bio-Rad, Richmond, CA, USA), followed by the addition of 100 μL of recombinant peptide (0.6 μg/mL; Alpalife, Shenzhen, Guangdong, China), corresponding to the extracellular region of GluN1 (N21–Q559). After the peptide solution was completely filtered through the membrane, the wells were blocked with 100 μL of 2% bovine serum albumin (BSA; 155681, Jackson ImmunoResearch, West Grove, PA, USA) in TBS-0.05% Tween-20 (TBST) at room temperature until the blocking solution was drained. Afterward, 20 μL of pooled CSF, 100 μL of serum (1:1000 in TBST), 100 μL of anti-GluN1 antibody (serves as a positive control, 1:600 dilution in TBST, AGC-001, Alomone), or 100 μL of normal mouse IgG (serves as a negative control, 1:1000 dilution in TBST; 61656, CST) was added onto the membrane and incubated for 1 h at room temperature. The wells were then washed 3 times with 400 μL of TBST using the vacuum source. Subsequently, the wells were incubated with 200 μL of HRP-conjugated anti-human IgG secondary antibody (1:10,000; bs-0297G-HRP, Bioss, Shanghai, China), anti-rabbit IgG secondary antibody (1:2000; 111-035-003, Jackson ImmunoResearch), or anti-mouse IgG secondary antibody (1:10,000; 115-035-003, Jackson ImmunoResearch) for 1 h at room temperature. Membrane was removed from the apparatus and washed with TBST three times (10 min each). Dots were visualized with Immobilon Western chemiluminescent HRP substrate (WBKLS0500, Merck, Darmstadt, German) and images were acquired with ImageQuant LAS 4000 (GE Healthcare Life Sciences, Issaquah, WA, USA).

### Immunofluorescence assay

Tissues were embedded in the optimal cutting temperature (OCT) compound and sectioned at 100 µm or 20 µm with a cryostat. Frozen sections were permeabilized and blocked in PBS containing 5% normal goat serum (SL038, Solarbio) and 0.3% Triton X-100 (V900502, Vetec) for 1 h at room temperature. The sections were then incubated with primary antibody against CD4 (1:50; ab288724, Abcam), CD8 (1:100; ab237709, Abcam), B220 (1:100; 14-0452-86, eBioscience), Laminine (1:100; MA106100, Invitrogen), P-Glycoprotein (1:72; MA1-26528, Invitrogen), GFAP (1:400; 3670S, CST), Il-1β (1:200; P420B, Invitrogen), or Fibrinogen (1:100; NBP2-80414, Novus) overnight at 4 °C. After 3 washes in PBS, sections were incubated with Alexa Fluor^®^ 488-conjugated anti-rabbit IgG (H + L) (1:500; 4412S, CST) and Alexa Fluor^®^ 555-conjugated anti-rat IgG (H + L) (1:500; 4417S, CST), or with Alexa Fluor^®^ 594-conjugated anti-mouse IgG (H + L) (1:500; 8890S, CST), for 1 h at room temperature. In addition, DAPI (1:2000; Sigma, 32670) was used to stain cell nuclei. Slides were mounted with Fluoro-Gel (17985-10, Electron Microscopy Sciences, Hatfield, PA, USA) and imaged using an Olympus IX71 fluorescence microscope (Olympus).

### Western blot

Total proteins (20 μg/lane) were separated using sodium dodecyl sulfate–polyacrylamide gel electrophoresis (SDS–PAGE) and transferred to a polyvinylidene difluoride (PVDF) membrane. The PVDF membrane was then blocked with 5% nonfat dry milk in TBST for 2 h at room temperature, incubated with primary antibodies overnight at 4 °C, and then incubated with secondary antibody for 1 h at room temperature. The bands were visualized with Immobilon Western chemiluminescent HRP substrate, and images were obtained with ImageQuant LAS 4000. The ImageJ software was used to quantify the band intensity, and GAPDH, β-Tubulin, or α-Actinin protein levels were used for normalization in their related Western blot experiments. The information for the antibodies in the Western blot experiments is collected in Additional file 1: Table S5.

### RNA-Seq analysis

The brain tissues were collected for RNA-Seq analysis. Total RNAs were extracted with TRIzol reagent (15596026, Invitrogen,), and RNA quality was assessed using NanoDrop ND-1000 (Thermo Fisher Scientific, Wilmington, DE, USA). RNA integrity was assessed by Bioanalyzer 2100 (Agilent, Landenberg, PA, USA). RNA samples (RNA concentration > 50 ng/μL, RNA integrity number (RIN) > 7.0) were used to construct cDNA libraries and sequence using a paired-end method, according to a standardized procedure, on the Illumina NovaseqTM 6000 platform (Lianchuan Biotechnology Co. Ltd., Hangzhou, Zhejiang, China). The raw reads were trimmed with adaptors and low-quality reads (such as sequence with a high content of unknown bases) were removed with FastQC v 0.11.9. The clean reads were then aligned to the *Mus musculus* reference genome (GRCm38) by HISAT v 2.2.0 [25], followed by calculation of the raw counts at the gene level using a GTF file with comprehensive gene annotation on the reference chromosomes (GRCm38) in the featureCounts program [26]. The raw counts were then used to identify differentially expressed genes (DEGs), with the cutoff of |Log2 fold change|> 1 and adjusted *p* < 0.05, by the DESeq2 v 1.30.1 R package. DEG-based method and Gene Set Enrichment Analysis (GSEA) were applied for gene ontology annotation using the biological process (GOBP) database. For the DEG-based method, commonly (between patient *vs.* HC and patient *vs.* vehicle) upregulated and downregulated genes were analyzed separately on Metascape using default settings [27]. Similar terms were clustered together and a representative (summarized) term (Log10(q) <  − 1.3013) from each cluster was plotted on a bar chart using ggplot2 R packages. The network of enriched terms with Log10(q) less than − 1.3013 was constructed and colored by clusters using Cytoscape software (version 3.9.1). For GSEA, genes were ranked according to the values calculated by − log10(padj)*sign(log2FoldChange) and used for analysis of GOBP gene sets (minSize = 10, maxSize = 800) with the fgsea R package. The potential correlations among the 417 common DEGs was searched using the online STRING tool (http://string-db.org) with medium confidence > 0.4. The protein–protein interaction (PPI) network was then built by Cytoscape software. The cytoHubba plugin and the “degree method” were used to identify the top 5 hub genes.

### Bulk RNA-Seq data deconvolution analysis

The cell type proportions of each sample were estimated from bulk RNA-Seq data using the deconvolution method provided by MuSiC R package [28]. For single-cell reference, we used single-cell transcriptomic analysis of young mouse brains downloaded from Gene Expression Omnibus (GSE129788). A total of 16,028 cells were identified as 6 major cell types, including neurons (NEUR), astrocytes (ASC), immune cells (IMMUNE), endothelial progenitor cells (EP), oligodendrocyte (OLG), and vascular endothelial cells (VASC), according to the metadata reported by the original study [29]. The code for deconvolution followed the standard tutorial at https://xuranw.github.io/MuSiC/articles/MuSiC.html.

### Real-time quantitative polymerase chain reaction (RT-qPCR)

RT-qPCR was performed to verify the differential expression of genes. Total RNAs were extracted with TRIzol reagent and 1 μg RNA was loaded for cDNA synthesis using HiScript III RT SuperMix (R323-01, Vazyme, Nanjing, China). Expression of genes was determined using TransStart^®^ Tip Green qPCR SuperMix (AQ141, Transgen, Beijing, China) on the ABI StepOne Plus Real-Time PCR system (Applied Biosystems, CA). Relative fold changes of genes to the control group were calculated using the 2^−ΔΔCT^ method. Fold changes were scaled across groups and are shown in the heatmap. Primers are listed in Additional file 1: Table S6.

### In vitro model of blood–brain barrier (BBB)

Cell culture inserts (353096, Falcon, Kennett Square, PA, USA) with 3 μm pores and a surface area of 0.3 cm^2^ were coated with 0.2% gelatin (G0040, solarbio) on both sides and placed in the 24-well cell culture insert companion plate (3,533,504, Falcon). The human astroglia cell line SVG p12 (1.2 × 10^5^ cells; ATCC^®^ CRL‐8621™) was obtained from Hunan Fenghui Biotechnology Co., Ltd. (Changsha, Hunan, China) and seeded on the underside of the insert and cultured in Dulbecco’s Modified Eagle Medium (DMEM; C1995500BT, Gibco, Franklin, TN, USA) supplemented with 10% fetal bovine serum (FBS; ST30-3302, PAN, Cologne, Germany), 100 IU/mL penicillin (15140122, Gibco), and 100 μg/mL streptomycin (15140122, Gibco). The hCMEC/D3 brain endothelial cells (1.2 × 10^5^ cells; SCC066) were purchased from EK-Bioscience (Shanghai, China), seeded to the upper side of the insert, and cultured in endothelial cell medium (ECM; 1001, Sciencell, Carlsbad, CA, USA). The medium was changed every 2–3 days. Cultures were maintained in 5% CO_2_ at 37 °C.

### Trans-endothelial electrical resistance (TEER) measurement

The TEER of in vitro BBB model was determined using the Millicell ERS-2 voltohmmeter (Merck). Briefly, the medium was removed from the upper chamber and the insert was transferred to a new 24 well containing 0.9 mL of fresh DMEM medium supplemented with FBS, penicillin, and streptomycin. Then, 0.3 mL of fresh ECM medium was added to the upper chamber and the electrodes were placed in the upper and lower chambers to measure the electrical resistance value (Ω). TEER was expressed in Ω × cm^2^ and the fold change in TEER relative to baseline was calculated.

### FITC–dextran permeability assay

The permeability of in vitro BBB model was assessed using 4 kDa FITC–dextran tracer solution (100 μg/mL). The insert was transferred to a new 24-well plate containing 0.6 mL of fresh DMEM medium supplemented with FBS, penicillin, and streptomycin. The medium in the upper chamber was replaced by 100 μL of FITC–dextran solution. After 3 h, the fluorescence in the lower chamber was measured by a multimode microplate reader (Victor Nivo 5S, Perkin Elmer, Greenville, SC, USA). A positive control was prepared by adding 100 μL of FITC–dextran directly into the lower chamber. The medium without cell culture and tracer served as a negative control. The percent fluorescence recovery was calculated as follows: [fluorescence (lower chamber) − fluorescence (negative control)]/[fluorescence (positive control) − fluorescence (negative control)] × 100%.

### Cell transfection with Il-1β-overexpression plasmid and siRNA

For plasmid transfection, cells were seeded and grown to 80% confluency. Empty (2.5 μg) or Il-1β-overexpression plasmid (2.5 μg, CH842401, Wzbio, Shandong, China) was mixed with 5 μL of P3000™ (L3000015, Invitrogen, CA, USA) and 125 μL of Opti-MEM medium (31985070, Gibco, CA, USA). Then, the mixture was mixed with 125 μL of Opti-MEM medium containing 5 μL of Lipofectamine™ 3000 (L3000015, Invitrogen, CA, USA) and incubated for 15 min at room temperature. The mixture was added to the cell culture medium. Cells were cultured for an additional 48 h before further treatment. For siRNA transfection, cells were seeded and grown to 40% confluency. siRNA oligos (50 nM) was diluted in 125 μL of Opti-MEM medium and mixed with 5 μL of Lipofectamine™ 3000 in 125 μL of Opti-MEM medium. After 15 min incubation, the mixture was added to the cell culture medium. Cells were cultured for an additional 48 h before further treatment. The negative control siNC (siN0000001-1-5) and IL-1β-specific siRNA oligos were obtained from RiboBio Co., Ltd. (Guangzhou, China). The target sequences of sill-1β were as follows: sil, 5′CGATGCACCTGTACGATCA-3, and si2, 5GATGTCTGGTCCATATGAA-3.

### Anakinra treatment

To block IL-1β-mediated effect, anakinra (ANK), a clinically used IL1 receptor antagonist protein, was applied in vitro and in vivo. For the in vitro BBB model, ANK was added directly to the cell at the final concentration of 500 ng/mL. For the intervention study, mice were intraperitoneally injected with 100 μL of ANK or vehicle at 10 mg/kg/day for 8 weeks from the day of PBMC engraftment.

### Statistical analysis

Shapiro–Wilk test was applied to test for data normality. To compare the means of two groups, Student's *t* test was used if the data were normally distributed; otherwise, Mann–Whitney *U* test was performed. For comparison of more than two groups, one-way analysis of variance (ANOVA) with post hoc Tukey’s tests were used. Statistical power analysis was used to validate the small sample size (*n* = 3) for significant differences (≥ 0.8 for sufficiently power validation), which was implemented in the freeware GPower [30]. A *p* value or adjusted *p* value less than 0.05 was considered statistically significant. For the analysis of structural MRI, the sizes of brain regions were compared between different groups using sklearn to conduct the Mann–Whitney *U* test, and the brain regions with significantly different volumes were highlighted in the standard template. For the analysis of ALFF, the paired *t* test (*p* < 0.05, cluster size ≥ 10) under null hypothesis was used to compare.

## Results

### Patient PBMC-humanized mice elicited autoantibody production and hippocampal GluN1 loss in *BRGSF* mice

**Fig. 2:**
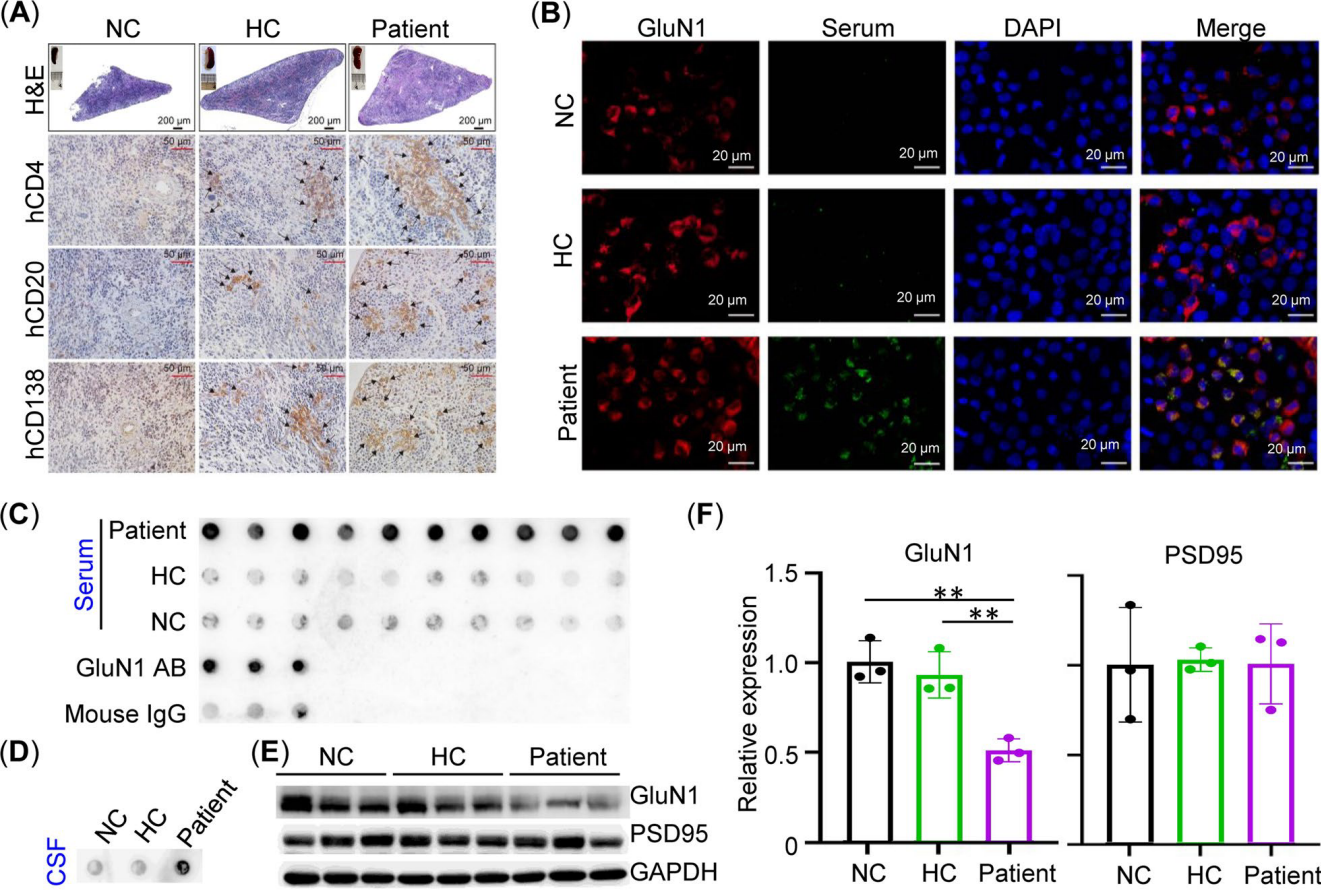
Production of autoantibody against GluN1 and hippocampal GluN1 loss in patient PBMC-humanized mice. **A** Representative micrographs of H&E stainings and immunohistochemical stainings of hCD4, hCD20, and hCD138 in splenic sections of BRGSF mice injected with medium (NC) or PBMCs (HC or patient) (*n* = 3/group). **B** Immunofluorescence of M68CT–GluN1 in HEK-293T cells (*n* = 3/group). Red, signals detected by commercial anti-GluN1 antibody. Green, signals detected by mouse serum from different groups. Nuclei were counterstained with DAPI (blue). **C** Dot blot using serum from mice (*n* = 10/group) to detect the extracellular region of GluN1 (N21–Q559). Commercial GluN1 antibody (AB) and mouse IgG were used as positive control and negative controls (*n* = 3), respectively. **D** Dot blot using pooled CSF from mice (pooled from 10 mice/group) to detect the extracellular region of GluN1 (N21–Q559). **E** Western blot of hippocampal GluN1 and PSD95 from NC group, HC group, and patient-group mice (*n* = 3/group). **F** Statistical analysis of relative protein expression in **E**. **p < 0.01, one-way ANOVA with Tukey’s post hoc test

Eight weeks after injection, no animals had died due to the PBMCs transfer. The engrafted human lymphocytes could be determined in the peripheral blood, bone marrow (BM), and spleen of the PBMC-humanized mice, as indicated by the presence of CD45^+^, CD4^+^, CD8^+^, and CD19^+^ human leukocytes on flow cytometry (Fig. 1B). Notably, the percentage of CD4^+^ T cells among the mCD45^−^hCD45^+^hCD3^+^ cells in the BM of the patient group was lower than that of the HC group (*n* = 3, *p* < 0.01), while an opposite trend was observed in the spleen between the groups (*n* = 3, *p* < 0.05, Additional file 1: Figure S1A). On the other hand, compared to the HC group, the percentage of CD8^+^ T cells among the mCD45^−^hCD45^+^hCD3^+^ cells was higher in the BM of the patient group (*n* = 3, *p* < 0.01) but lower in the spleen of the patient group (*n* = 3, *p* < 0.01, Additional file 1: Figure S1A). There was no significant difference in the percentage of CD19^+^ B cells among the mCD45^−^hCD45^+^hCD3^−^ cells between the patient group and the HC group (*n* = 3, Additional file 1: Figure S1B). Histologically, CD4^+^ T cells, CD20^+^ B cells, and CD138^+^ plasma cells were observed in the spleens of all PBMC-humanized mice, but not in NC-group mice (Fig. 2A). Of note, transfer of PBMC restored the splenic white pulp in the immunodeficient mice, suggesting that human leukocytes formed germinal center-like structures (Fig. 2A). Given that infused human lymphocytes survived and formed splenic white pulp, we explored whether antibodies against NMDAR could be produced in the recipient mice. Immunostaining of GluN1 (subunit of NMDAR)-transfected HEK-293 T cells and dot blot were used. As shown in Fig. 2, the antibody against GluN1 in the mouse serum of the patient group tested positive in both the immunofluorescence (Fig. 2B) and the dot blot assay (Fig. 2C, D), while it was absent in the serum from the NC or HC group (Fig. 2C). Due to the limited volume from a mouse, we pooled the CSF from all mice and detected the antibody using the dot blot assay. Consistent with the serum, autoantibodies against GluN1 were positive in the CSF of the patient group, but not in the NC and HC group (Fig. 2D). Furthermore, Western blot showed that mice transferred with patient PBMCs had a lower level of hippocampal GluN1 compared with NC-group mice and HC–PBMC-transferred mice (*n* = 3, Fig. 2E, F). PSD95 proteins were not altered between groups. These results suggested that anti-NMDAR antibodies were present in the humanized mice and might be associated with the reduction in GluN1 protein levels in brain tissue.

### Transfer of patient PBMC altered behaviors and induced structural and functional changes in recipient mice

**Fig. 3:**
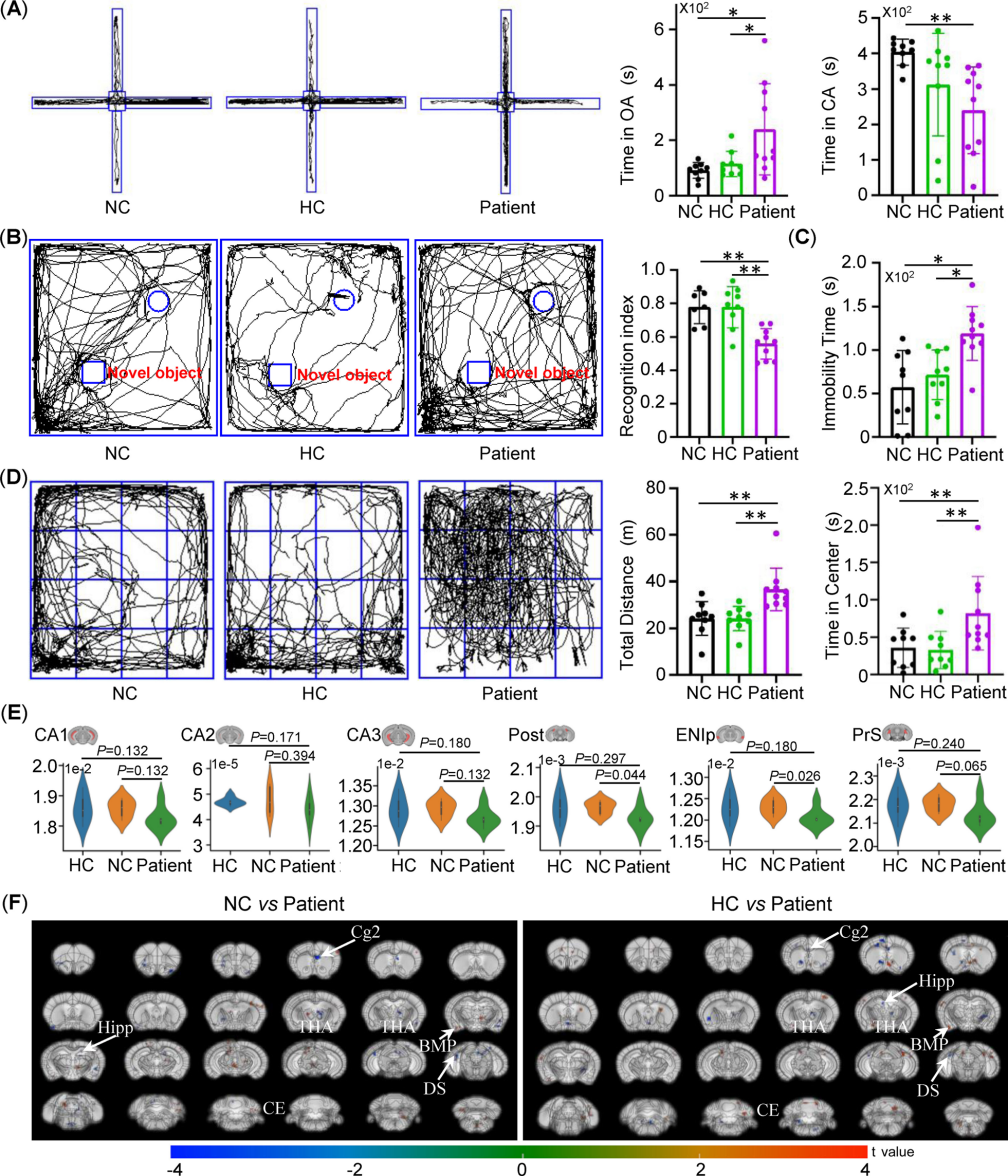
Abnormal animal behaviors, and structural and functional changes in different brain regions of the patient PBMC-humanized mice. **A** Representative movement traces of NC-group mice (*n* = 9), HC-group mice (*n* = 9), and patient-group mice (*n* = 10) in the elevated plus maze (EPM) to examine claustrophobia-like behavior. **B** Representative movement traces of NC-group mice (*n* = 7), HC-group mice (*n* = 9), and patient-group mice (*n* = 10) in the novel object recognition test (NORT) to examine memory-learning deficits. **C** Immobility time of the animals in the NC group (*n* = 9), HC group (*n* = 9), and patient group (*n* = 10) in forced swimming test to assess depressive-like behavior. **D** Representative movement traces of the animals in the NC group (*n* = 9), HC group (*n* = 9), and patient group (*n* = 10) in the open-field test to examine hyperactive locomotor and anxiety behaviors. **E** Comparisons of volumes of different brain regions between groups. **F** Comparisons of ALFF values between the NC groups vs. patient group and HC vs. patient group (*n* = 6/group). **p < 0.01, one-way ANOVA with post hoc Tukey’s test

Patients with anti-NMDAR encephalitis may present with psychiatric symptoms, cognitive deficits, and abnormal movements [4]. To assess whether PBMC engraftment could recapitulate neuropsychiatric disorders in patients, we used a battery of standardized tests. BRGSF mice from three groups, as above, were examined for behavioral changes 8 weeks after vehicle or PBMC injection. In the elevated plus maze test, the mice transferred with patient PBMCs spent more time in the open arms and less time in the closed arms than NC-group mice and mice carrying HC–PBMC, suggesting a claustrophobia-like behavior accompanied by anti-NMDAR autoantibody production (Fig. 3A). Meanwhile, cognition was measured using novel object recognition. Compared to the mice from the NC group and the HC group, mice transferred with patient PBMCs had a lower discrimination index in the novel object recognition test (Fig. 3B), suggesting deficits in memory and learning. In addition, mice from the patient group also exhibited depressive-like behaviors, as indicated by the longer periods of immobility than NC-group mice and HC–PBMC-treated mice in the forced swimming test (Fig. 3C). This was not caused by suppression of locomotion; instead, open-field testing confirmed a hyperactive locomotor phenotype in patient-group mice, as evidenced by the increased total distance traveled compared to NC-group mice and HC-group mice (Fig. 3D). There were no differences in any behaviors between NC mice and mice transferred with HC–PBMC. Collectively, these data suggested that transfer of patient PBMC could alter neuropsychiatric behavior in humanized mice*.*

We then explored whether changes in neuropsychiatric behavior were associated with changes in volumetric structure and intrinsic cerebral functions in different brain regions. Voxel-based morphometry (VBM) and amplitude of low-frequency fluctuation (ALFF) analysis were performed on mice 8 weeks after vehicle or PBMC injection (Fig. 1A). We found that there was no significant difference in the volumes of most brain regions, including various hippocampal subregions, between the groups (Fig. 3E). However, the patient group had a lower volume in the lateral entorhinal area (*p* = 0.026, NC vs. patient, 1.23 × 10^–2^ ± 1.87 × 10^–4^ mm^3 vs.^ 1.21 × 10^–2^ ± 2.08 × 10^–4^ mm^3^) and post subiculum (*p* = 0.045, NC vs. patient, 1.96 × 10^–3^ ± 2.28 × 10^–5^ mm^3^ vs. 1.93 × 10^–3^ ± 3.44 × 10^–5^ mm^3^) than the NC group. There was no significant difference between HC and patient group (lateral entorhinal area: *p* = 0.179; post subiculum: *p* = 0.297).

On the other hand, transfer of PBMCs from patients altered the activities of multiple brain regions. Compared to the mice from the NC group and the HC group, mice with engrafted patient PBMCs had increased ALFF values in the hippocampus, thalamus, prefrontal cortex, cingulate cortex, area 2 (Cg2), and DS (dorsal subiculum) (Fig. 3F, corrected *p* < 0.05 between NC and patient or between HC and patient) but reduced ALFF values in several other regions, such as the BMP (basomedial amygdaloid nucleus, posterior part) and CE (cerebellum) (Fig. 3F, corrected *p* < 0.05 between groups). We propose that transfer of patient PBMCs modulated cerebral function but not structures, and that abnormal mental processing could be attributed to neuropsychiatric disorders in mice with patient-related immune systems.

Acute graft vs. host disease (GVHD), frequently reported during the application of PBMC-humanized mice models, is attracting attention due to the risk of misinterpretation of disease models [31, 32]. In the current study, mice transferred with a high dose of cells (1 × 10^7^ cells) from healthy or patient donors all presented evident signs of GVHD such as weight loss and compromised skin integrity. This was prevented by lowering cell counts as recommended by a previous study. Transfer of PBMCs at 1 × 10^6^ cells did not show any signs of GVHD during the 8-week experimental period.

### Humanized patient–PBMC mice exhibited a leaky BBB

**Fig. 4:**
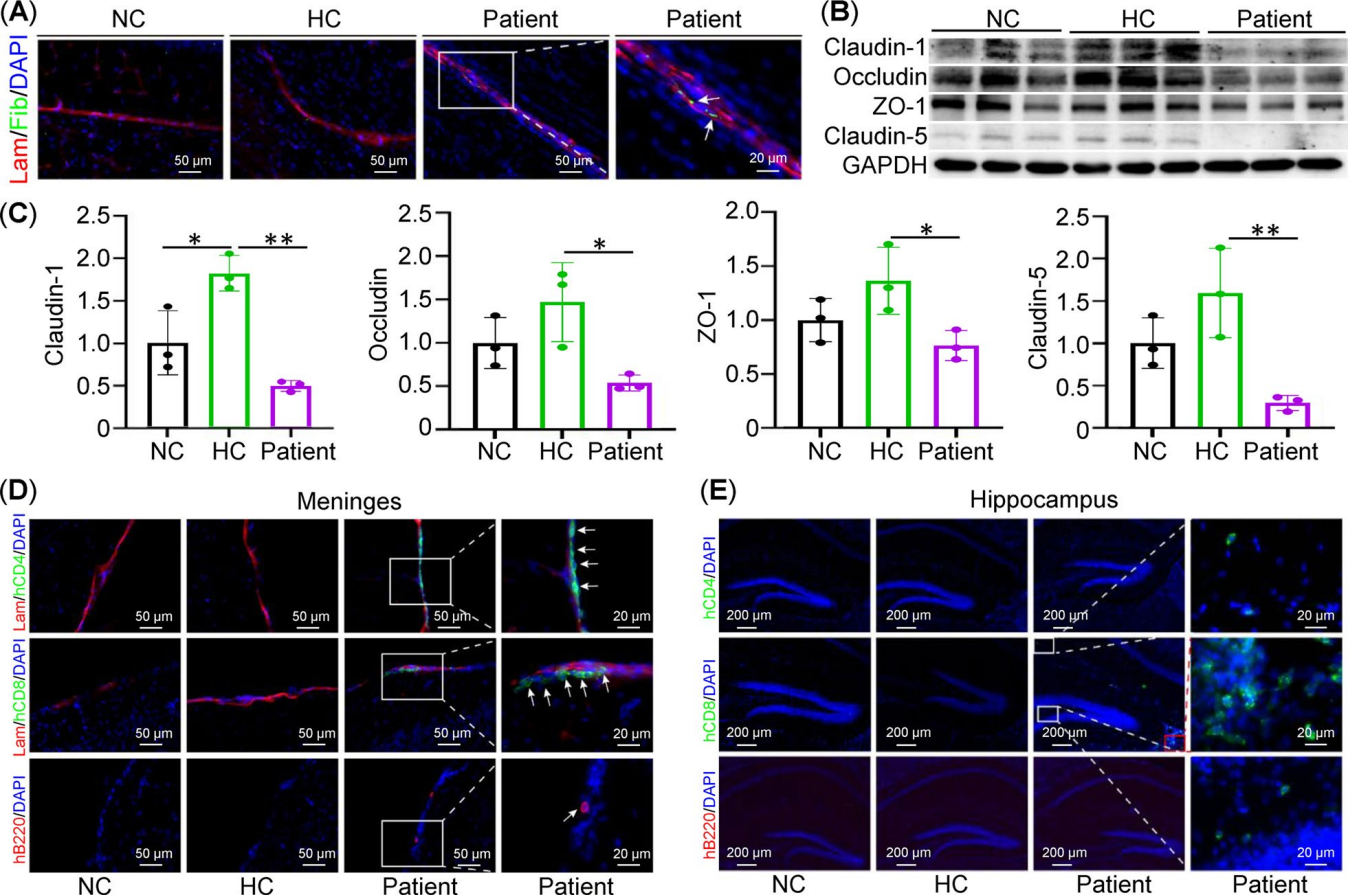
Leaky BBB in patient PBMC-humanized mice. **A** Immunofluorescence of laminin (Lam; vessels, red) and fibrinogen (Fib; index of BBB leakage, green) in mouse brain (*n* = 3/group). Nuclei were counterstained with DAPI (blue). **B** Western blot of tight junction proteins (Claudin-1, Occludin, ZO-1, and Claudin-5) on total tissue lysates from whole brain (*n* = 3/group), respectively. **C** Statistical analysis of the relative protein expression in **B**. **D** Immunofluorescence of laminin (Lam; vessels, red) and human CD4 (CD4^+^ lymphocytes, green), or human CD8 (CD8^+^ lymphocytes, green), or B220 (B lymphocytes, red) in meninges of mice (*n* = 3/group). Nuclei were counterstained with DAPI (blue). **E** Immunofluorescence of human CD4 (green), human CD8 (green), or B220 (red) in hippocampus (*n* = 3/group). Nuclei were counterstained with DAPI (blue)

Patients with anti-NMDAR encephalitis are characterized by elevated cytokines in CSF and immune cell infiltration [33], which have been associated with a damaged BBB in patients. Interestingly, in our humanized PBMC mice models, fibrinogen extravasation was observed in the brains of mice from the patient group but not from the NC and HC groups, indicative of a leaky BBB in mice transferred with patient PBMCs (Fig. 4A). Tight junction proteins, such as Occludin, Claudin-1, Claudin-5, and ZO-1, are the essential components that connect brain microvascular endothelial cells (BMVECs) and seal the intracellular gaps between BMVECs to maintain the integrity of the BBB. Consistent with fibrinogen extravasation, transfer of PBMCs from patients reduced the protein levels of Claudin-1, Occludin, ZO-1, and Claudin-5 compared to HC–PBMC injection (Fig. 4B, C). Moreover, this was accompanied by infiltrations of CD4^+^ T cells, CD8^+^ T cells, and B220^+^ B cells in meninges (Fig. 4D), and of CD8^+^ T cells in the hippocampus (Fig. 4E) in mice injected with patient PBMCs. No immune cell infiltration was observed in mice from the NC group and the HC group. These results suggested that patient-derived PBMCs were involved in BBB leakage, leading to immune cell entry into the CNS through tight junction protein suppression.

### Endothelial Il-1β damaged BBB in humanized patient–PBMC mice

**Fig. 5:**
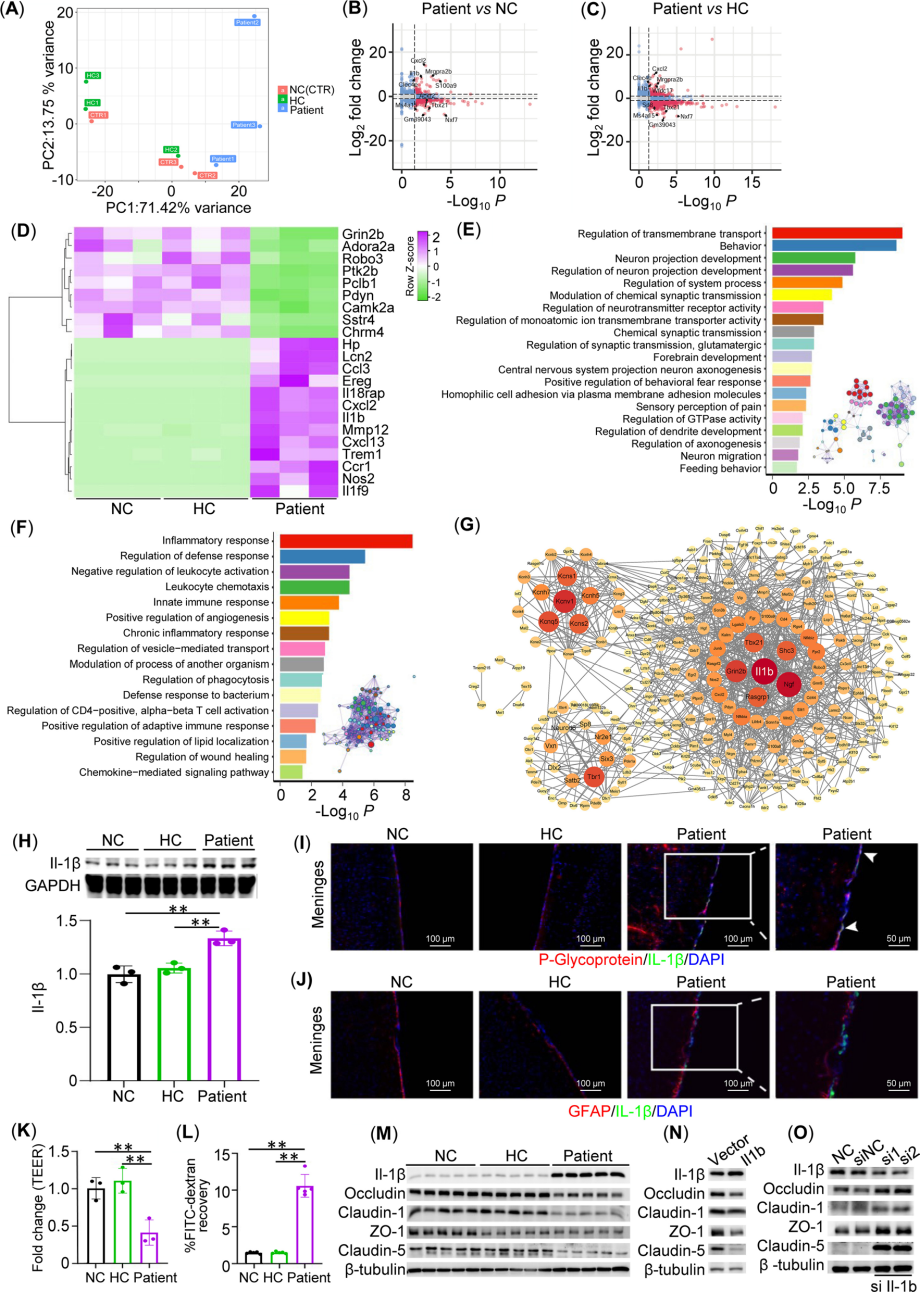
Endothelial Il-1β damaged BBB in patient PBMC-humanized mice. **A** PCA showing a clear overall deference in the transcriptome between the patient group and the HC (healthy control) and Ctrl (medium control) group. **B, C** Volcano plot showing DEGs between groups (*n* = 3/group). **D** RT-qPCR validation of DEGs in **B** and **C**. **E** Bar chart of 20 summarized GOBP terms (adjusted *p* < 0.05) based on the commonly (between patient vs. HC and patient vs. NC) downregulated DEGs and interaction network of all significant GOBP terms. Nodes refer to GOBP terms with similar semantics as summarized terms in the same color. **F** Bar chart of 16 summarized GOBP terms (adjusted *p* < 0.05) based on the commonly upregulated DEGs and interaction network of all significant GOBP terms. **G** PPI network of common DEGs. **H** Western blot of IL-1β on total tissue lysates from whole brain (*n* = 3/group) and statistical analysis. **I** Immunofluorescence of P-Glycoprotein (brain microvascular endothelial cells, red) and Il-1β (green) in meninges (*n* = 3/group). Nuclei were counterstained with DAPI (blue). **J** Immunofluorescence of GFAP (astrocytes, red) and Il-1β (green) in meninges (*n* = 3/group). Nuclei were counterstained with DAPI (blue). **K** Relative changes in transendothelial electrical resistance (TEER) of in vitro BBB model with different treatments (*n* = 5/group). **L** FITC-dextran recovery of in vitro BBB model with different treatments (*n* = 5/group). **M** Western blot of Il-1β, Occludin, Claudin-1, ZO-1, and Claudin-5 on cell lysates from hCMEC/D3 cells with different treatments (*n* = 5/group). **N** Western blot of IL-1β, Occludin, Claudin-1, ZO-1, and Claudin-5 on cell lysates from hCMEC/D3 cells transfected with empty vector or Il-1β-overexpression plasmid (*n* = 3). **O** Western blot of Il-1β, Occludin, Claudin-1, ZO-1, and Claudin-5 on cell lysates from hCMEC/D3 cells transfected with medium, siRNA control (siNC), or Il1b siRNA (si1 and si2) (*n* = 3). **p < 0.01, one-way ANOVA with post hoc Tukey’s test

To explore the potential molecular mechanism underlying BBB leakage, we conducted transcriptome analysis. Mice were randomly assigned to 3 groups (*n* = 3 per group) as above and treated accordingly. Eight weeks after medium or PBMC injection, brains were collected for bulk RNA-Seq analysis. Principal component analysis (PCA) showed a clear overall difference in the transcriptome between patient group and HC or control group (Fig. 5A). Further DEG analysis revealed 417 common DEGs (fold change > 2) between patient group vs. NC group (490 DEGs, Fig. 5B) and patient group vs. HC group (1268 DEGs, Fig. 5C). The differential expression of 22 common DEGs was validated by quantitative RT-PCR (Fig. 5D). DEG-based (Fig. 5E, F) and GSEA-based (Additional file 1: Figure S2) methods were used for gene ontology annotation, which highlighted impaired neurotransmission but exaggerated inflammatory responses in mice with anti-NMDAR encephalitis. Terms related to the nervous system are enriched in the list of downregulated genes (Fig. 5E) or have NES < 0 in GSEA (Additional file 1: Figure S2B). In addition, *cell junction organization* (NES = − 1.698, adjusted *p* = 3.28E-4, Additional file 1: Figure S3) and *cell junction assembly* (NES = − 1.56, adjusted *p* = 2.37E-10) might explain the BBB disruption from the perspective of transcriptomic change. On the other hand, it is noted that upregulated DEGs are involved in various pathways related to the immune system (Fig. 5F, Additional file 1: Figure S2A), suggesting dramatic immunological changes. The protein–protein interaction (PPI) network of 417 common DEGs was retrieved from the STRING database. It was noted that Il-1β was the gene with the highest degrees in the complete network, and it was identified as the hub gene of a significant molecular complex detection (MCODE) module (Fig. 5G and Additional file 1: Figure S5).

Il-1β has been demonstrated to be closely involved in BBB disruption in many studies [34–37]. Meanwhile, it was shown that Il-1β could increase intestinal permeability by repressing the expression of tight junction genes, including Occludin [38]. Thus, we investigated the role of Il-1β in the pathogenesis of encephalitis in humanized PBMC mice. Consistent with the transcriptome result, transfer of patient PBMCs resulted in an elevated protein levels of Il-1β compared to NC group or HC–PBMC group (Fig. 5H). We then examined the sources of elevated Il-1β by immunofluorescence. P-Glycoprotein-labeled microvascular endothelial cells (Fig. 5I), specifically, but not astrocytes (Gfap^+^, Fig. 5J), were contained with anti-Il-1β, indicating that patient PBMCs could upregulate endothelial Il-1β to damage the BBB. This was supported by findings from the in vitro BBB model. Compared to HC control, treatment with PBMCs from patients reduced the TEER in the hCMEC/D3 microvascular endothelial cell line and enhanced its permeability to dextran (Fig. 5K, L). In accordance with in vivo findings, patient PBMCs also directly upregulated protein levels of Il-1β in hCMEC/D3 cells while decreasing Claudin-1, Occludin, ZO-1, and Claudin-5 (Fig. 5M, Additional file 1: Figure S6A). Interestingly, we further found that the disrupted tight junction in endothelia cells was Il-1β-dependent. Overexpression of Il-1β in hCMEC/D3 cells decreased the protein levels of Claudin-1, Occludin, ZO-1, and Claudin-5 (Fig. 5N, Additional file 1: Figure S6B), while knockdown of Il-1β displayed an opposite effect (Fig. 5O, Additional file 1: Figure S6C). Taken together, we propose that patient PBMCs could directly upregulate endothelial Il-1β, which decreases tight junctions in brain microvascular endothelial cells, leading to BBB leakage.

### Blocking Il-1β signaling ameliorates autoimmune encephalitis in patient PBMC-humanized mice

**Fig. 6:**
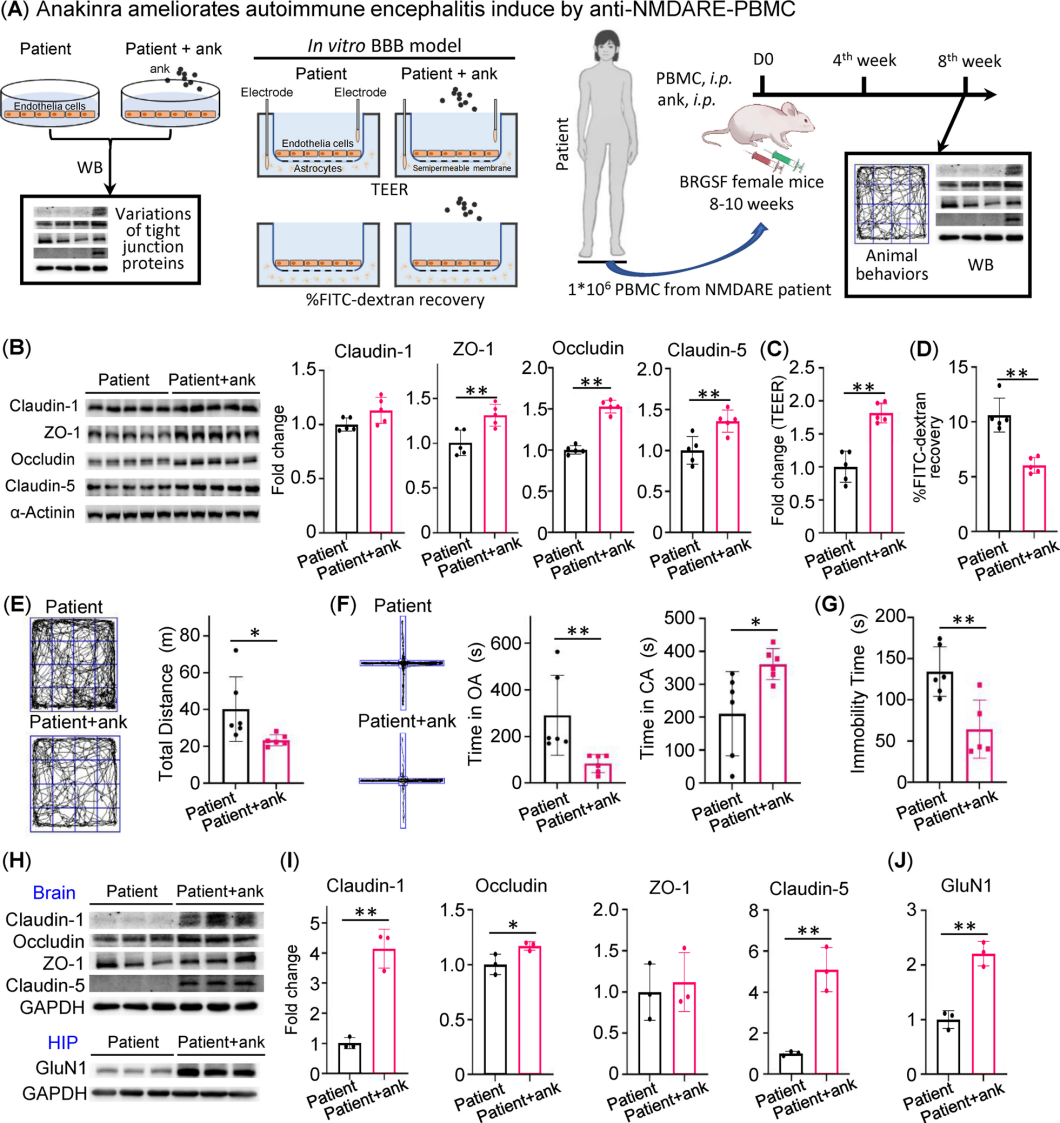
Anakinra (ank) ameliorates autoimmune encephalitis in patient PBMC-humanized mice. **A** Schematic diagram of the experimental design. **B** Western blot of Claudin-1, Occludin, ZO-1, and Claudin-5 on cell lysates from hCMEC/D3 cells (*n* = 5/group) and statistical analysis of relative protein expression. **C** Relative changes in transendothelial electrical resistance (TEER) of in vitro BBB model (*n* = 5/group). **D** FITC–dextran recovery of in vitro BBB (*n* = 5/group). **E** Representative movement traces of patient-group mice (*n* = 6) and ank-treated patient + ank group mice (*n* = 6) in the open-field test. **F** Representative movement traces of mice from different groups (*n* = 6) in the elevated plus maze (EPM). **G** Immobility time of mice from different groups (*n* = 6) in the forced swimming test. **H** Western blot of tight junction proteins (Claudin-1, Occludin, ZO-1, and Claudin-5) on total tissue lysates from whole brains (*n* = 3/group). Western blot of hippocampal GluN1 (*n* = 3/group). **I** Statistical analysis of relative protein expression in **H**. **J** Statistical analysis of relative protein expression in **H**. *p < 0.05, **p < 0.01, Student’s *t* test

We then examined the therapeutic value of Il-1β blockage in the humanized mouse model of anti-NMDAR encephalitis. Anakinra, an Il-1 receptor antagonist protein approved for the treatment of rheumatoid arthritis, was used to block Il-1β signaling. The schematic diagram of the experimental design is shown in Fig. 6A. Anakinra reversed patient PBMC-induced suppression of Occludin, ZO-1, and Claudin-5 in hCMEC/D3 cells (Fig. 6B). Accordingly, compared to vehicle control, treatment with Anakinra enhanced TEER while reducing the permeability to FITC–dextran in hCMEC/D3 cells in the presence of patient PBMCs (Fig. 6C, D). Anakinra was also effective in vivo. Mice were administered Anakinra or vehicle for 8 weeks after PBMC injection. Compared to vehicle, Anakinra improved hyperactive locomotor behaviors, claustrophobia-like behaviors, and depressive-like behaviors (Fig. 6E–G). At the molecular level, Anakinra reversed the suppression of Claudin-1, Occludin, and Claudin-5 (Fig. 6H, I) in the whole brain and restored the protein levels of GluN1 in the hippocampus (Fig. 6H, J). These results suggested that Anakinra ameliorated autoimmune encephalitis by restoring BBB permeability.

## Discussion

**Fig. 7:**
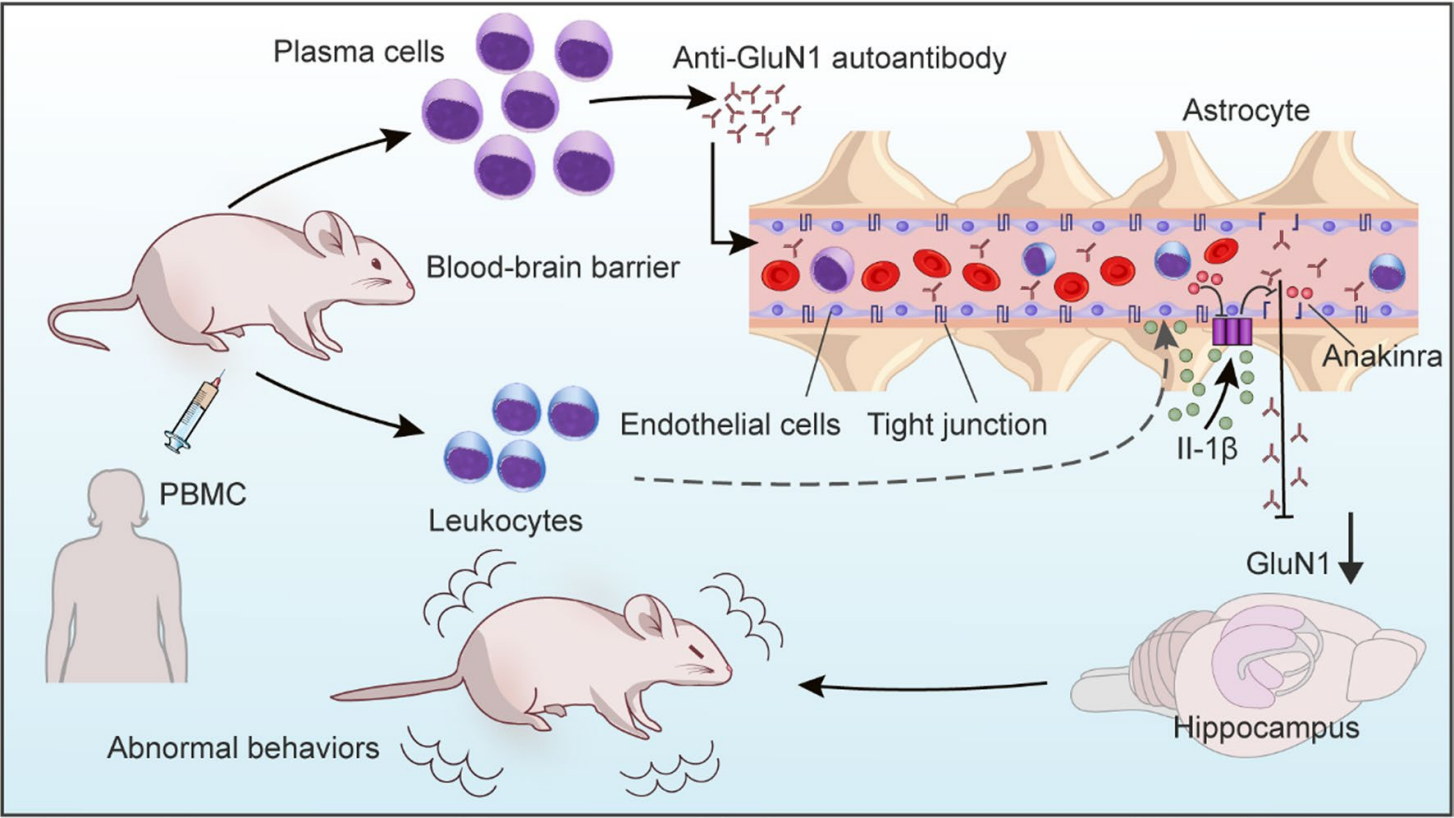
Illustration of the major findings in the current study

In the current study, we developed a humanized mouse model of anti-NMDAR encephalitis by transferring PBMCs from patients to BRGSF mice. Engraftment of patient lymphocytes induced anti-GluN1 autoantibody production and access to the brain, promoted lymphocyte infiltration, and caused abnormal neuropsychiatric behaviors as well as functional changes in multiple brain regions associated with neuropsychiatric behaviors. We highlighted BBB leakage as a critical pathological change in a humanized mouse model and identified endothelial Il-1β as a hub DEG that could disrupt the BBB by downregulating tight junction proteins in the endothelium. Moreover, we demonstrated that blocking Il-1β receptor by Anakinra reversed BBB leakage, immune cell infiltration, and neuropsychiatric disorders in a humanized mouse model of anti-NMDA encephalitis (Fig. 7). Our study provided a clinically relevant model of anti-NMDAR encephalitis and emphasized blocking Il-1β signaling as a promising strategy to ameliorate autoimmune encephalitis.

The humanized model could be adopted to investigate the pathogenic role of BBB damage in patients with anti-NMDAR encephalitis. The pathogenesis of anti-NMDAR encephalitis requires two major components. First, B cells are abnormally activated by the self-GluN1 subunit of NMDAR and then differentiated into effector cells for the production and secretion of autoantibody in the serum. This is followed by autoantibody access to the brain, where anti-GluN1 autoantibodies cause a selective and reversible decrease in the synaptic localization of NMDARs, leading to the pathogenesis of cognitive deficits and neuropsychiatric symptoms observed in patients [39]. Several routes for entry of antibodies into the CNS have been proposed, including the blood–cerebrospinal fluid barrier (BCSFB), olfactory route, and BBB [40]. Notably, the BBB appears to be an important route for anti-NMDAR autoantibody access, as BBB damage is associated with disease severity and intrathecal IgG synthesis in patients with anti-NMDAR encephalitis [41]. In fact, bypassing the BBB is essential for the development of previous animal models of anti-NDMAR encephalitis. In the passive transfer model, the patient-derived anti-NMDAR antibody is delivered directly to the brain by intraventricular injection [6], while coadministration with pertussis toxin, which disrupts the BBB, is a prerequisite for the active immunization model [7]. In this regard, previous models may neglect the pathogenic role of BBB disruption in this autoimmune encephalitis. It is also impossible to pinpoint the etiology of BBB breakdown using these models. In the current humanized mouse model, production of human anti-NMDAR antibody was detected in the serum of BRGSF mice, suggesting that the patient-derived immunized B cell subpopulation is proliferating and functioning normally, a requisite for a successful modeling. Moreover, the human anti-NMDAR antibody is also dramatically elevated in mouse CSF, accompanied by a decreased hippocampal NMDAR protein in the recipient mice. These results implied that the anti-NMDAR autoantibodies enter the CNS efficiently, without artificially disrupting BBB, and that these autoantibodies detected in the humanized mice are functional and similar to the patient-derived anti-NMDAR antibodies [6, 39]. The current model provides a more clinically relevant framework to study the role of BBB damage in the pathogenesis of anti-NMDAR encephalitis in vivo.

The humanized mouse model suggested a pathogenic role of PBMCs in anti-NMDAR encephalitis. Unlike previous models that use pertussis toxin or genetic manipulation (Apoe knockout) to disrupt the BBB, a simple transfer of PBMCs mirrors patients’ condition by exhibiting a donor-dependent BBB permeability, implying that patient-derived lymphocytes might be detrimental to BBB integrity and pathogenic. In stroke models, it is demonstrated that infiltrating T lymphocytes (e.g., Th17 and Th1 cells) might regulate cytokines (e.g., Infγ and Il-17), chemokines (Ccl2 and Cxcl1), or ROS to degrade tight junction in the BBB [42]. It should be noted that these T lymphocytes may not directly secrete BBB-damaging pro-inflammatory mediators but, instead, amplify inflammatory cascade by remodeling gene expression in cells within the BBB. In the current humanized model, we also observed brain infiltration of Cd4^+^ and Cd8^+^ T lymphocytes only in mice transferred with patient PBMCs, a potential link to BBB dysfunction. This was further supported by the in vitro assay, which demonstrated decreased TEER, increased permeability to dextran, and reduction in tight junction proteins in the endothelial cell line by direct incubation with purified patient PBMCs. Therefore, in addition to autoantibody synthesis (B cells), the model reveals a new pathogenetic role of lymphocyte subpopulations by directly perturbing the BBB. It is likely that certain T cell subpopulations might be educated to initialize the cascade of pathogenesis. The current model represents a valuable tool to enrich our understanding of lymphocyte subpopulations attributed to anti-NMDAR encephalitis-associated BBB disruption in vivo.

Transcriptome analysis identified IL-1β as a potential hub DEGs. It is notable that IL-1β was one of the most upregulated genes in mice with anti-NMDAR encephalitis. Moreover, PPI analysis highlighted IL-1β as a hub in the whole network and in a highly interconnected subnetwork, implying that Il-1β could act as a regulator driving disease-associated changes in the gene network. Il-1β is a robust cytokine that might elicit strong immune cascade, including induction of chemokines (such as MIP-2) for the recruitment of immune cells’ infiltration into the CNS [43–47]. Il-1β can be synthesized by various cell types in the CNS, depending on models of neurological disorders. For example, astrocytes and microglia are demonstrated to secrete abundant Il-1β in models of multiple sclerosis (MS) [48]. In this study, interestingly, we found that Il-1β could be produced by the endothelial cells in vivo and in vitro in response to patient PBMCs. Elevated Il-1β causes a reduction in tight junction proteins (Claudin-1, Occludin, ZO-1, and Claudin-5) and an increase in BBB permeability. These results indicated that Il-1β is a potential target to treat anti-NMDAR encephalitis. Indeed, Anakinra, an Il-1 receptor antagonist, was demonstrated to maintain BBB integrity and ameliorate behavioral disorders associated with autoimmune encephalitis.

This study has some limitations. Transfer of PBMCs to immunodeficient mice has been adopted in several humanized models of autoimmune encephalitis. In this study, we reported a similar humanized mouse model of anti-NMDAR encephalitis that can partially recapitulate human symptoms as in other passive animal models [39, 49, 50]. However, the species difference in immune genes can cause defects in the immune response of transplanted human cells and thus inevitably affect the pathogenesis of autoimmune diseases in humanized mice. For example, one of the most significant species differences between humans and rodents is the absence of FcγRIIA and FcγRIIC, both of which are activating Fc receptors, in mice [51]. Fc receptors are widely expressed in various immune cells and the balance between activating and inhibitory Fc receptors is crucial for maintaining the antibody-mediated responses. On B cells, FcγRIIB (an inhibitory Fc receptor) is the only Fc receptor, and knockout of FcγRIIB elicited systemic lupus erythematosus-like phenotypes and the production of autoantibodies in mice [52]. In the humanized mouse models, although human Fc receptors could be synthesized in transplanted immune cells, the expression level of Fc receptors might be altered in mice, resulting in unparalleled autoantibody production between patients and humanized mice. Second, it has been reported that the frequency of B cells is low in humanized mouse models including in those for SSc [16, 17] and Myasthenia Gravis [12]. Although in our and others’ models, we successfully detected autoantibodies that are associated with disease-related phenotypes, the insufficient antibodies might mask some of the symptoms or pathogenetic mechanism in patients. Finally, even though autoantibodies are detectable in the serum and CSF, there is a chance that the behavioral changes and other pathological changes are attributed to other cell populations than those of autoantibody-specific B cell lineage. Future work adopting selective depletion of B cells or T cells prior to injection or pharmacologically after injection would dissect the effects of the different cell-type populations.

To our knowledge, this is the first PBMC transfer-induced humanized mouse model for anti-NMDAR encephalitis. Using the new animal model, we proposed that patient-derived PBMC might trigger encephalitis pathogenesis by causing Il-1β-dependent BBB dysfunction, and that the IL1 receptor antagonist Anakinra is a potential treatment for anti-NMDAR encephalitis. The new animal model expands our potential to directly evaluate the influence of cellular and humoral functions in the progression of this autoimmune encephalitis. In addition, the easy-to-handle model makes it practical to establish individual patient-specific mouse models for the evaluation of various potential therapies or the development of precision medicine.

## Supplementary Information

**Additional file 1.** Supplemental tables and figures.

**Additional file 2.** The demographic and clinical characteristics of the subjects were collected.

## Data Availability

The data in this study are available from the corresponding author upon reasonable request.
