# Supplementary material for: Transfer of patient’s peripheral blood mononuclear cells (PBMCs) disrupts blood–brain barrier and induces anti-NMDAR encephalitis: a study of novel humanized PBMC mouse model
Source: J Neuroinflammation. 2023 Jul 13;20:164. doi: 10.1186/s12974-023-02844-4 (PMC10339507; doi:10.1186/s12974-023-02844-4)
Supplement: Supplementary file 1 — Additional file 1. Supplemental tables and figures. [file 12974_2023_2844_MOESM1_ESM.docx]

**Additional materials**

Table S1. Demographic and clinical features of healthy donors and anti-NMDARE patients for the in vivo animal experiment.

| Group | Anti-NMDARE patients | | | | | | | | | Healthy controls | | | | | | | | |
| --- | --- | --- | --- | --- | --- | --- | --- | --- | --- | --- | --- | --- | --- | --- | --- | --- | --- | --- |
| Subject | 1 | 2 | 3 | 4 | 5 | 6 | 7 | 8 | 9 | 1 | 2 | 3 | 4 | 5 | 6 | 7 | 8 | 9 |
| Age | 22 | 22 | 26 | 19 | 30 | 18 | 33 | 16 | 20 | 32 | 28 | 20 | 25 | 28 | 30 | 37 | 27 | 23 |
| Gender | F | F | F | F | F | M | F | F | F | M | F | F | M | F | F | M | F | M |
| mRS initial admission | 4 | 4 | 5 | 5 | 5 | 4 | 5 | 4 | 5 | / | / | / | / | / | / | / | / | / |
| mRS at discharge | 1 | 2 | 3 | 4 | 2 | 3 | 4 | 2 | 2 | / | / | / | / | / | / | / | / | / |
| Serum anti-NMDARabs titers | 1:10 | 1:100 | 1:32 | 1:100 | 1:100 | 1:32 | 1:32 | 1:10 | 1:100 | / | / | / | / | / | / | / | / | / |
| CSF anti-NMDARabs titers | 1:32 | 1:100 | 1:100 | 1:100 | 1:100 | 1:10 | 1:32 | 1:10 | 1:100 | / | / | / | / | / | / | / | / | / |
| Cognitive decline | - | + | + | - | + | + | + | + | - | / | / | / | / | / | / | / | / | / |
| Psychiatric symptoms | + | + | + | + | + | + | + | + | + | / | / | / | / | / | / | / | / | / |
| Seizures | + | - | + | + | + | - | + | + | + | / | / | / | / | / | / | / | / | / |
| Motor disturbance | - | - | - | - | + | - | + | - | + | / | / | / | / | / | / | / | / | / |
| Central hypoventilation | - | - | - | + | - | - | + | - | + | / | / | / | / | / | / | / | / | / |
| Involuntary movement | - | + | - | + | + | + | + | - | + | / | / | / | / | / | / | / | / | / |
| Abnormal MRI | + | - | - | + | - | - | - | + | - | / | / | / | / | / | / | / | / | / |
| Tumors | - | - | - | + | + | - | - | + | + | / | / | / | / | / | / | / | / | / |
| Response to treatments | Yes | Yes | Yes | Partial | Yes | Yes | Partial | Yes | Yes | / | / | / | / | / | / | / | / | / |

*Note: F: female, M: male; MRI: magnetic resonance imaging; mRS: Modified Rankin Scale; /: not relevant.*

**Table S2**. Demographic and clinical features of healthy donors and anti-NMDARE patients for the in vitro experiment.

| Group | Anti-NMDARE patients | | | | | Healthy controls | | | | | |
| --- | --- | --- | --- | --- | --- | --- | --- | --- | --- | --- | --- |
| Subject | 4 | 10 | 11 | 12 | 13 | 7 | 10 | 11 | 12 | 13 |  |
| Age | 19 | 18 | 19 | 23 | 22 | 38 | 26 | 25 | 30 | 26 |  |
| Gender | F | F | F | M | M | M | F | F | F | F |  |
| mRS initial admission | 5 | 4 | 3 | 4 | 4 | / | / | / | / | / |  |
| mRS at discharge | 4 | 3 | 1 | 1 | 2 | / | / | / | / | / |  |
| Serum anti-NMDARabs titers | 1:100 | 1:30 | 1:10 | 1:10 | 1:10 | / | / | / | / | / |  |
| CSF anti-NMDARabs titers | 1:100 | 1:30 | 1:10 | 1:10 | 1:32 | / | / | / | / | / |  |
| Cognitive decline | - | + | + | - | - | / | / | / | / | / |  |
| Psychiatric symptoms | + | + | + | + | + | / | / | / | / | / |  |
| Seizures | + | + | - | + | + | / | / | / | / | / |  |
| Motor disturbance | - | + | - | - | + | / | / | / | / | / |  |
| Central hypoventilation | + | + | - | - | + | / | / | / | / | / |  |
| Involuntary movement | + | + | - | - | - | / | / | / | / | / |  |
| Abnormal MRI | + | - | - | - | - | / | / | / | / | / |  |
| Tumors | + | - | - | - | - | / | / | / | / | / |  |
| Response to treatments | Partial | Partial | Yes | Yes | Yes | / | / | / | / | / |  |

*Note: F: female, M: male; MRI: magnetic resonance imaging; mRS: Modified Rankin Scale; /: not relevant. Subjects 4 and 7 were also utilized to withdraw the blood samples for the in vivo animal experiments.*

**Table S3.** Mice engrafted with human PBMCs from healthy controls and anti-NMDARE patients.

| Input human PBMCs | Injected PBMCs | Number of injected mice | Number of mice for behavioral assessments | Number of MRI-scanned mice | Number of mice for Anakinra |
| --- | --- | --- | --- | --- | --- |
| control #1 | Frozen | 1 | 0 | 0 | 0 |
| control #2 | Frozen | 1 | 0 | 0 | 0 |
| control #3 | Frozen | 1 | 0 | 0 | 0 |
| control #4 | Frozen | 6 | 5 | 3 | 0 |
| control #5 | Frozen | 2 | 2 | 2 | 0 |
| control #6 | Frozen | 2 | 2 | 1 | 0 |
| control #7 | Frozen | 1 | 0 | 0 | 0 |
| control #8 | Frozen | 1 | 0 | 0 | 0 |
| control #9 | Frozen | 1 | 0 | 0 | 0 |
| **Total** |  | 16 | 9 | 6 | 0 |
| patient #1 | Frozen | 2 | 0 | 0 | 0 |
| patient #2 | Frozen | 7 | 0 | 0 | 0 |
| patient #3 | Frozen | 1 | 0 | 0 | 0 |
| patient #4 | Frozen | 4 | 0 | 2 | 0 |
| patient #5 | Frozen | 4 | 3 | 2 | 0 |
| patient #6 | Frozen | 12 | 7 | 2 | 0 |
| patient #7 | Frozen | 9 | 7 | 0 | 4 |
| patient #8 | Frozen | 4 | 3 | 0 | 2 |
| patient #9 | Frozen | 2 | 2 | 0 | 1 |
| **Total** |  | **45** | **22** | **6** | **7** |

**Table S4**. Analysis of clinical characteristics of patients used for animal and in vitro study.

|  | **Animal study** | **In vitro study** | ***p*-values** |
| --- | --- | --- | --- |
| **Age** | 22.9±5.7 | 20.2±2.2 | 0.3361 |
| **Gender** |  |  |  |
| Female | 8 (88.9) | 3 (60.0) | 0.2418 |
| Male | 1 (11.1) | 2 (40.0) |  |
| **mRS at admission** | 5 (1) | 4 (0) | 0.1567 |
| **Serum titers** | 32 (90) | 10 (20) | 0.4016 |
| **CSF titers** | 100 (68) | 10 (20) | 0.1058 |
| **Cognitive decline** |  |  |  |
| Yes | 6 (66.7) | 2 (40.0) | 0.6084 |
| No | 3 (33.3) | 3 (60.0) |  |
| **Psychiatric symptoms** |  |  |  |
| Yes | 9 (100.0) | 5 (100.0) | 1 |
| No | 0 (0.0) | 0 (0.0) |  |
| **Seizures** |  |  |  |
| Yes | 7 (77.8) | 4 (80.0) | 1 |
| No | 2 (22.2) | 1 (20.0) |  |
| **Motor disturbance** |  |  |  |
| Yes | 3 (33.3) | 2 (40.0) | 1 |
| No | 6 (66.7) | 3 (60.0) |  |
| **Central hypoventilation** |  |  |  |
| Yes | 3 (33.3) | 3 (60.0) | 0.5804 |
| No | 6 (66.7) | 2 (40.0) |  |
| **Involuntary movement** |  |  |  |
| Yes | 6 (66.7) | 2 (40.0) | 0.5804 |
| No | 3 (33.3) | 3 (60.0) |  |
| **Abnormal MRI** |  |  |  |
| Yes | 3 (33.3) | 1 (20.0) | 1 |
| No | 6 (66.7) | 4 (80.0) |  |
| **Tumors** |  |  |  |
| Yes | 4 (44.4) | 1 (20.0) | 0.5804 |
| No | 5 (55.6) | 4 (80.0) |  |

*Note: The categorical data were presented as number (percentage) and compared between groups using Fisher’s exact test. Age was reported as mean±SD and compared between groups using Student’s t-test. mRS, serum, and CSF titers were reported as median (interquartile ranges) and compared between groups using the Wilcoxon rank sum test.*

**Table S5**. Collected information of the antibodies for Western blot experiment.

| Antibody | Company | Catalog # | Species | Dilution |
| --- | --- | --- | --- | --- |
| IL-1β | Invitrogen | P420B | Rabbit | 1:1000 |
| PSD95 | CST | 36233S | Mouse | 1:1000 |
| Occludin | CST | 91131S | Rabbit | 1:1000 |
| GluN1 | Invitrogen | 320500 | Mouse | 1:1000 |
| Claudin-1 | Proteintech | 13050 | Rabbit | 1:1000 |
| ZO-1 | Proteintech | 21773 | Rabbit | 1:5000 |
| Claudin-5 | Affinity | AF5216 | Rabbit | 1:1000 |
| GAPDH | CST | 5174S | Rabbit | 1:1000 |
| α-Actinin | CST | 69758S | Mouse | 1:1000 |
| HRP-conjugated anti-rabbit IgG secondary antibody | Jackson ImmunoResearch | 111-035-003 | Goat | 1:2000 |
| HRP-conjugated anti-mouse IgG secondary antibody | Jackson ImmunoResearch | 115-035-003 | Goat | 1:10000 |

**Table S6**. Collected information of the primers in the current study.

| Genes | Forward primer (5' to 3') | Reverse primer (5' to 3') |
| --- | --- | --- |
| Plcb1 | GCCCCTGGAGATTCTGGAGT | GGGAGACTTGAGGTTCACCTTT |
| Cxcl2 | CCAACCACCAGGCTACAGG | GCGTCACACTCAAGCTCTG |
| Il1b | GCAACTGTTCCTGAACTCAACT | ATCTTTTGGGGTCCGTCAACT |
| Cxcl3 | TCTCACCACAGCCCTTCGCA | AAAAACAAGCAGGTAAAGACACATC |
| Il1f9 | TCCTGACTTTGGGGAGGTTTT | TCACGCTGACTGGGGTTACT |
| Trem1 | GACTGCTGTGCGTGTTCTTTG | GCCAAGCCTTCTGGCTGTT |
| Ereg | CTGCCTCTTGGGTCTTGACG | GCGGTACAGTTATCCTCGGATTC |
| Lcn2 | TGGCCCTGAGTGTCATGTG | CTCTTGTAGCTCATAGATGGTGC |
| Ccl3 | TTCTCTGTACCATGACACTCTGC | CGTGGAATCTTCCGGCTGTAG |
| Hp | GCTATGTGGAGCACTTGGTTC | CACCCATTGCTTCTCGTCGTT |
| Mmp12 | CTGCTCCCATGAATGACAGTG | AGTTGCTTCTAGCCCAAAGAAC |
| Il18rap | AGACTACTTCCTGAGCACAAGA | TGTCCTTACCAATGGTTCTCACT |
| Nos2 | GTTCTCAGCCCAACAATACAAGA | GTGGACGGGTCGATGTCAC |
| Ccr1 | CTCATGCAGCATAGGAGGCTT | ACATGGCATCACCAAAAATCCA |
| Robo3 | GAGGCAGAGGGTTCTCTTACT | CGCCCCATTCTTGTACCACT |
| Camk2a | ACCTGCACCCGATTCACAG | TGGCAGCATACTCCTGACCA |
| Adora2a | GCCATCCCATTCGCCATCA | GCAATAGCCAAGAGGCTGAAGA |
| Grin2b | GCCATGAACGAGACTGACCC | GCTTCCTGGTCCGTGTCATC |
| Pdyn | CTCCTCGTGATGCCCTCTAAT | AGGGAGCAAATCAGGGGGT |
| Chrm4 | ATGGCGAACTTCACACCTGTC | CTGTCGCAATGAACACCATCT |
| Sstr4 | CAGCGGGCATGGTCACTATC | CCGTCCACGCTAAGCACTG |
| Ptk2b | ATCTTGACCACCCTCACATCG | TAGTGTCCCAGCTCCCCATAA |
| Gapdh | CCAGCAAGGACACTGAGCAA | GGGATGGAAATTGTGAGGGA |

**Supplemental figures:**


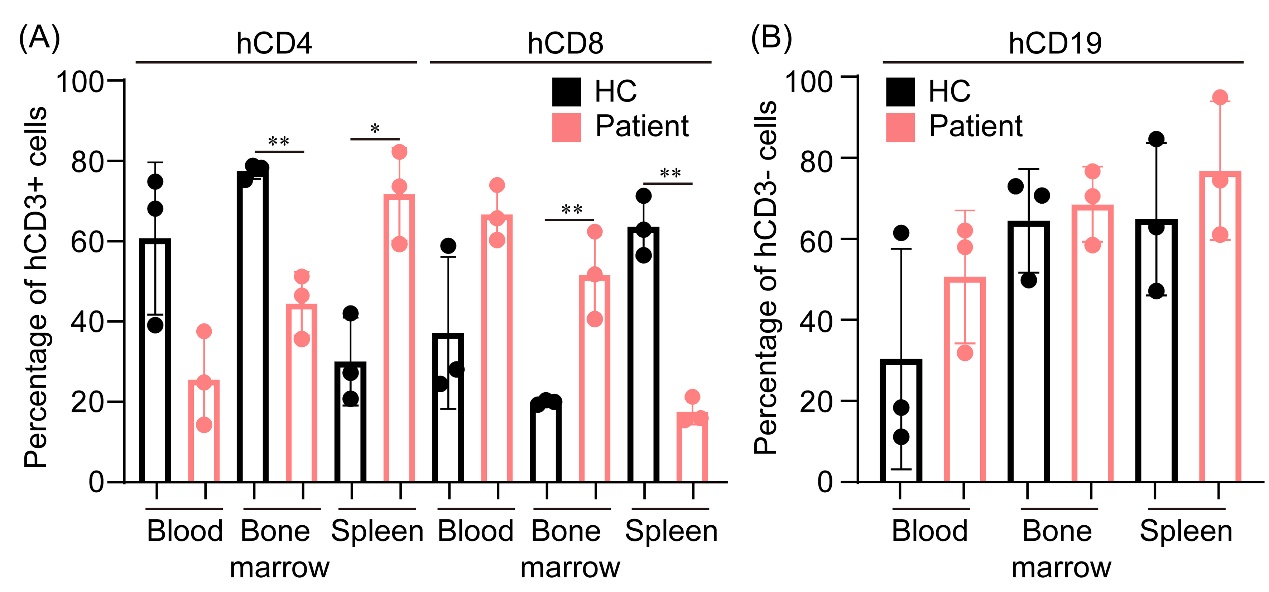


Figure S1. Statistical analysis of flow cytometry.

***A:*** *Flow cytometry of the percentage of human CD4^+^ and human CD8^+^ T cells in human CD3^+^ cells from the blood, bone marrow, and spleen of BRGSF mice injected with peripheral blood mononuclear cells (PBMCs) isolated from patients or HC subjects (n = 3/group).* ***B:*** *Flow cytometry of the percentage of human CD19^+^ B cells in human CD3- cells from the blood, bone marrow, and spleen of BRGSF mice injected with PBMCs isolated from patients or HC (n = 3/group). * p<0.05, ** p<0.01, Student’s t-test.*


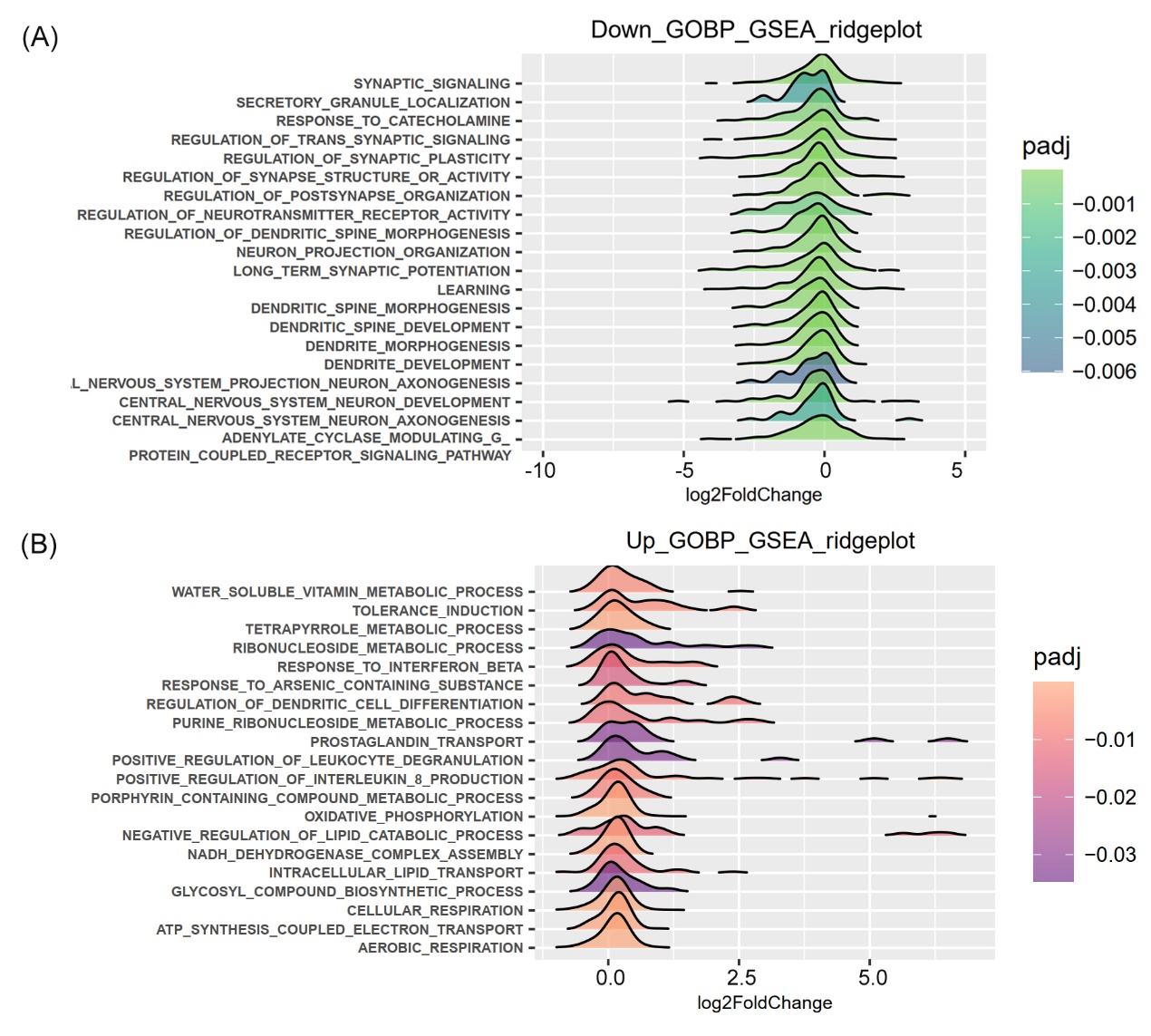


**Fig. S2**. GSEA of gene ontology.

***A and B****: The ridge-plot of top 20 GOBP terms with the lowest (A) or highest (B) normalized enrichment scores. The log2FoldChange refers to fold changes in genes of a GOBP term between patient group and HC group. All terms shown in the plots have an adjusted p-value less than 0.05.*

**
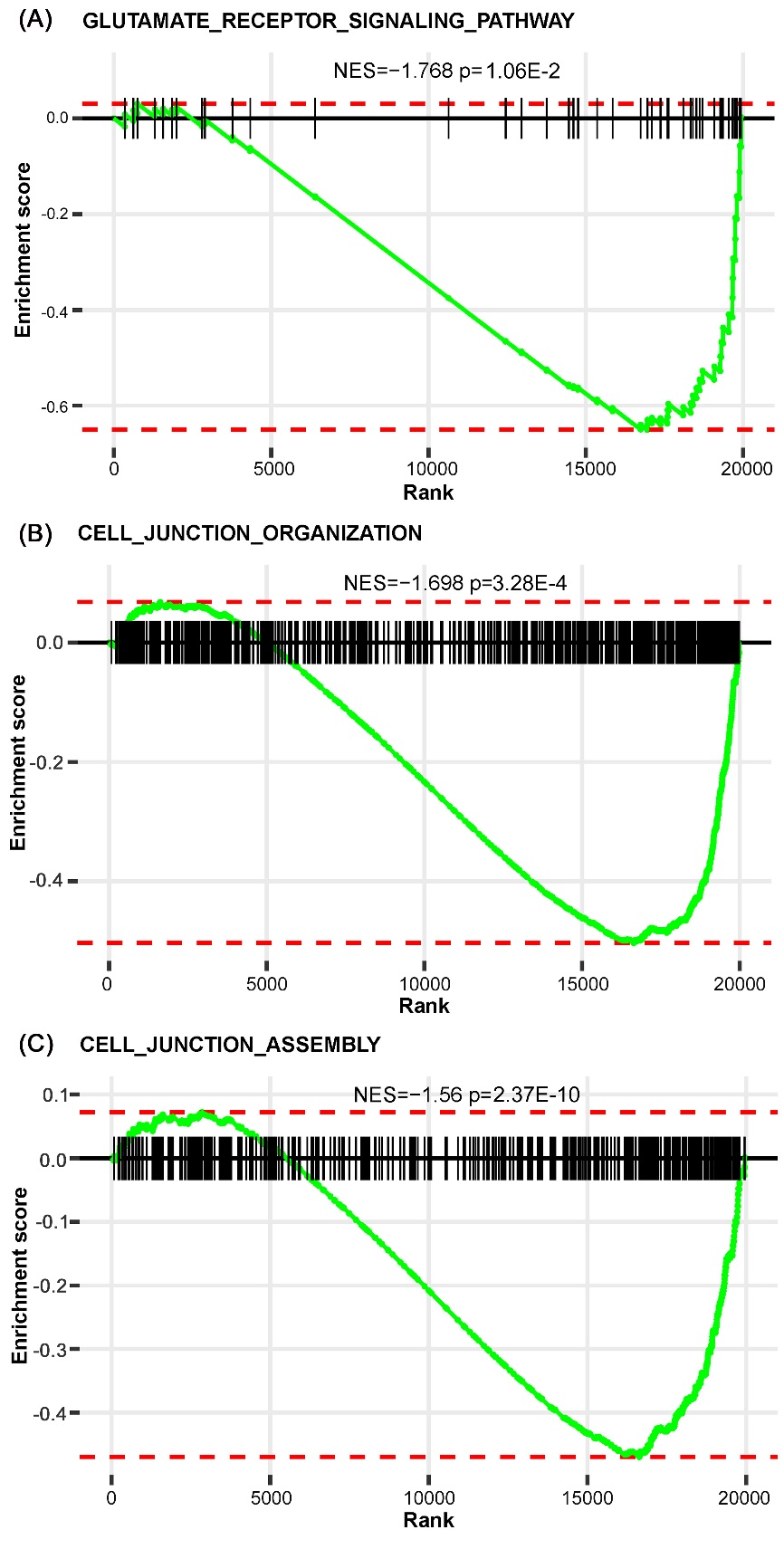
**

**Figure S3**. Representative enrichment plots from GSEA.

***A****:* *Glutamate receptor signaling pathway. B: Cell junction organization. C: Cell junction assembly.*

**
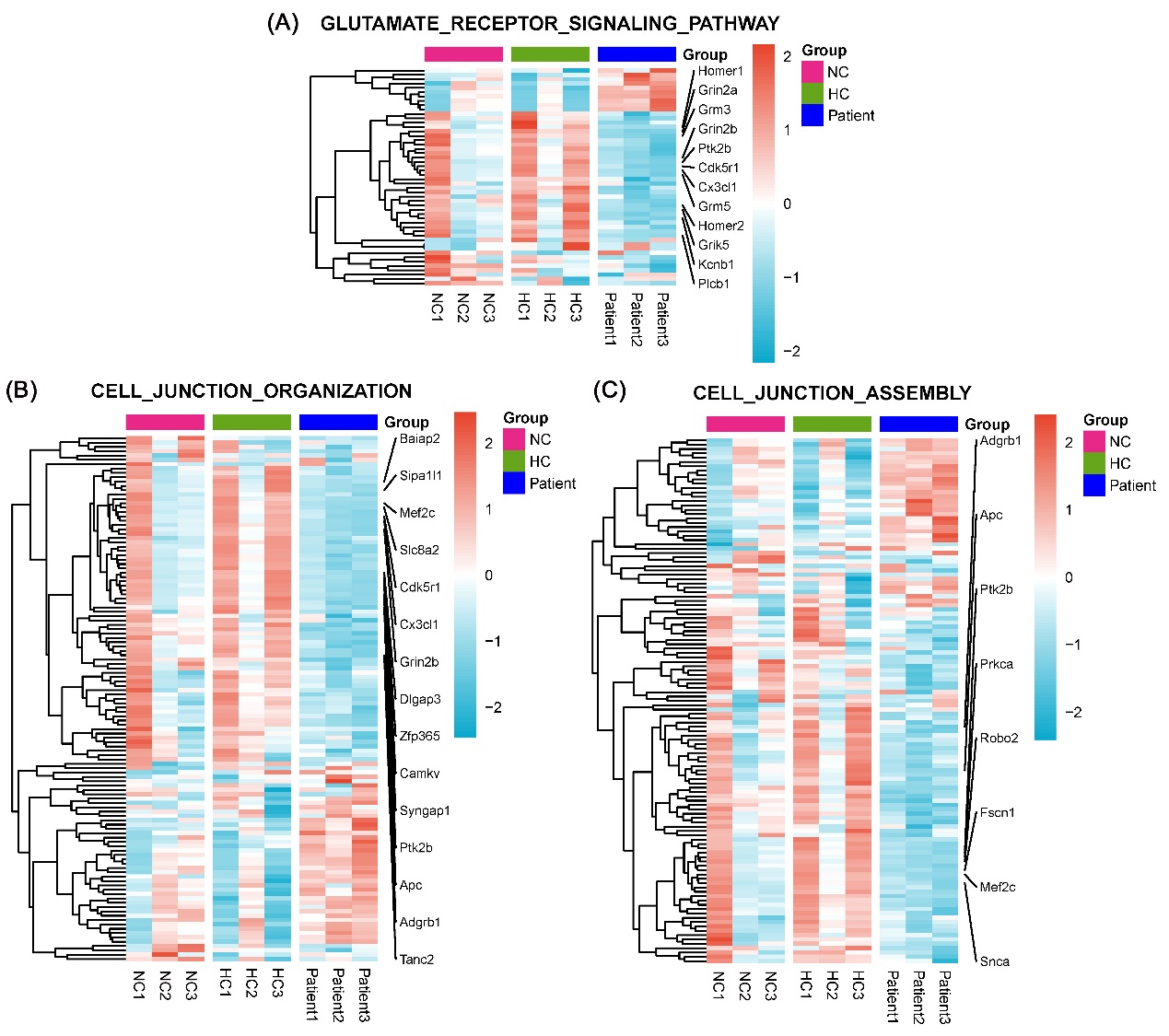
**

**Figure S4**. Representative heatmaps of GO terms illustrated in Fig. S3.

***A****,* ***B,*** *and* ***C****: Heatmaps of Glutamate receptor signaling pathway, cell junction organization, and cell junction assembly. The labeled genes are common DEGs between patient vs. HC and patient vs. NC.*


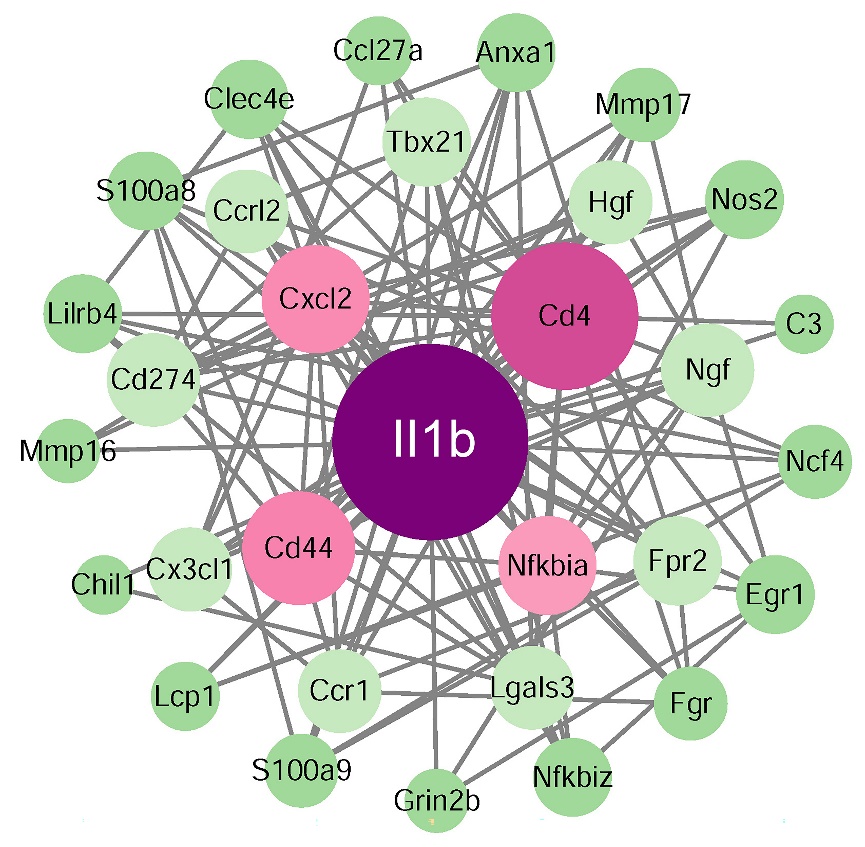


**Figure S5**. The highly interconnected module identified from the whole PPI network highlighted Il-1β as a hub gene.


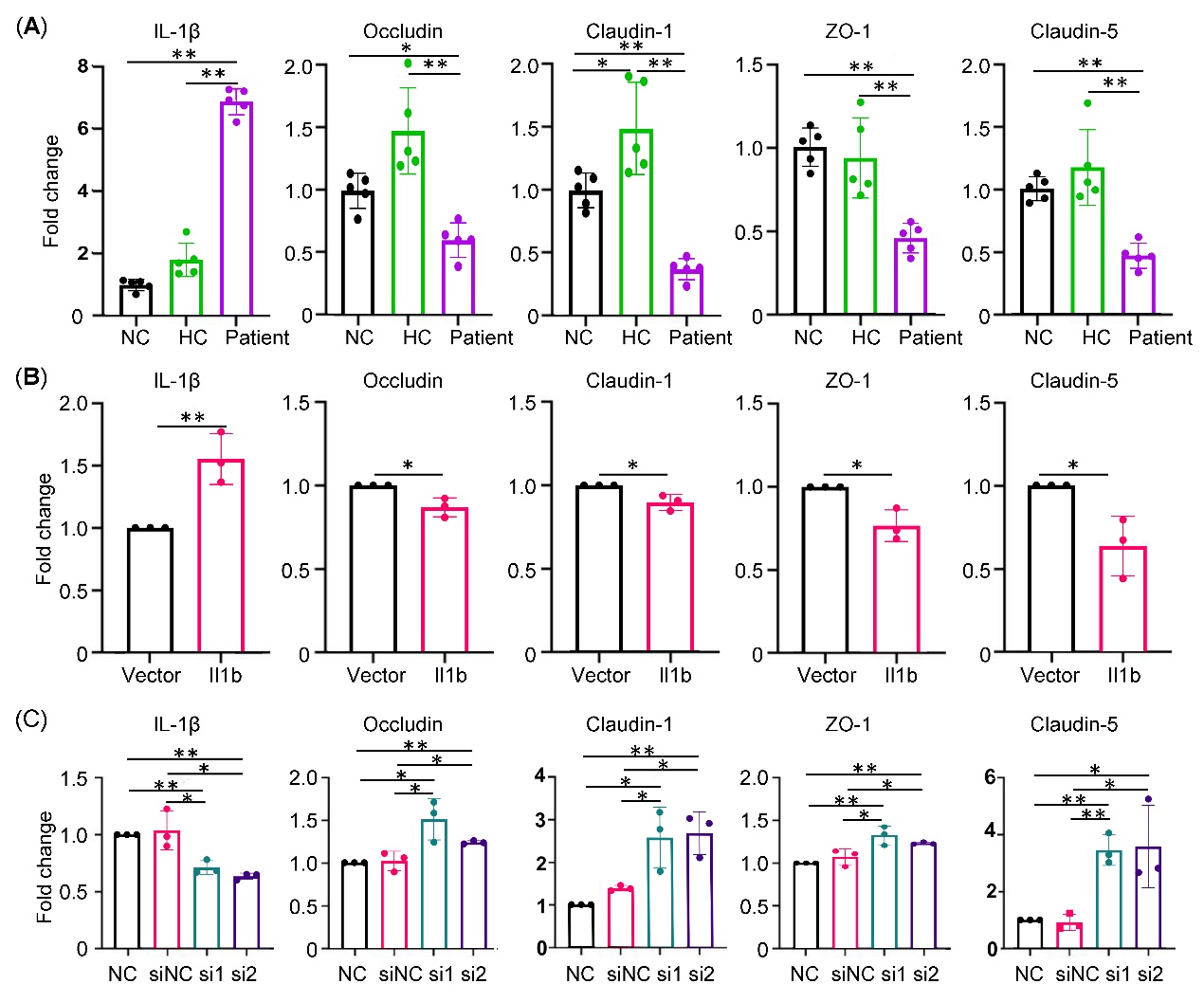


**Figure S6**. Statistical analysis of relative protein expression.

***A****: Statistical analysis of relative protein expression of Il-1β, Occludin, Claudin-1, ZO-1, and Claudin-5 on cell lysates from hCMEC/D3 cells with different treatments (n = 5). *p<0.05, **p<0.01, one-way ANOVA with post hoc Tukey’s test.* ***B****: Statistical analysis of relative protein expression of Il-1β, Occludin, Claudin-1, ZO-1, and Claudin-5 on cell lysates from hCMEC/D3 cells transfected with empty vector or Il-1β-overexpression plasmid (n = 3). *p<0.05, **p<0.01,* *Student’s t-test.* ***C****: Statistical analysis of relative protein expression of Il-1β, Occludin, Claudin-1, ZO-1, and Claudin-5 on cell lysates from hCMEC/D3 cells transfected with medium, siRNA control (siNC), or Il1b siRNA (si1 and si2) (n = 3).* **p<0.05, **p<0.01, one-way ANOVA with post hoc* *Tukey’s test.*
